# The logic of recurrent circuits in the primary visual cortex

**DOI:** 10.1038/s41593-023-01510-5

**Published:** 2024-01-03

**Authors:** Ian Antón Oldenburg, William D. Hendricks, Gregory Handy, Kiarash Shamardani, Hayley A. Bounds, Brent Doiron, Hillel Adesnik

**Affiliations:** 1 Department of Molecular and Cell Biology, University of California, Berkeley, Berkeley, CA USA; 2 The Helen Wills Neuroscience Institute, University of California, Berkeley, Berkeley, CA USA; 3 Department of Neurobiology and Statistics, University of Chicago, Chicago, IL USA; 4 Grossman Center for Quantitative Biology and Human Behavior, University of Chicago, Chicago, IL USA; 5 Present Address: Department of Neuroscience and Cell Biology, Robert Wood Johnson Medical School, and Center for Advanced Biotechnology and Medicine, Rutgers University, Piscataway, NJ USA; 6 Present Address: Department of Mathematics, University of Minnesota, Minneapolis, MN USA; 7 Present Address: Department of Neurology and Neurological Sciences, Stanford University, Stanford, CA USA

**Keywords:** Dynamical systems, Neural circuits, Striate cortex, Network models

## Abstract

Recurrent cortical activity sculpts visual perception by refining, amplifying or suppressing visual input. However, the rules that govern the influence of recurrent activity remain enigmatic. We used ensemble-specific two-photon optogenetics in the mouse visual cortex to isolate the impact of recurrent activity from external visual input. We found that the spatial arrangement and the visual feature preference of the stimulated ensemble and the neighboring neurons jointly determine the net effect of recurrent activity. Photoactivation of these ensembles drives suppression in all cells beyond 30 µm but uniformly drives activation in closer similarly tuned cells. In nonsimilarly tuned cells, compact, cotuned ensembles drive net suppression, while diffuse, cotuned ensembles drive activation. Computational modeling suggests that highly local recurrent excitatory connectivity and selective convergence onto inhibitory neurons explain these effects. Our findings reveal a straightforward logic in which space and feature preference of cortical ensembles determine their impact on local recurrent activity.

## Abstract (web summary)

Using two-photon (2P) optogenetics and computational modeling, the authors find that neither space-based nor feature-based rules are sufficient to describe cell–cell interactions within the primary visual cortex (V1). Instead, models must include interactions between these cardinal axes.

## Main

Visual perception involves the coordinated activity of thousands of neurons throughout the visual system. As the neural representation of sensory stimuli traverses each step of the visual hierarchy, recurrent circuits at each processing stage transform and refine it^1^. Prior experimental and theoretical work in the primary visual cortex (V1) suggests that recurrent excitation amplifies responses when signals are weak to optimize detection^2–6^, while recurrent inhibition suppresses responses when signals are strong to optimize discrimination^7–10^. Understanding what patterns of cortical activity drive either amplification or suppression is critical for a mechanistic understanding of signal transformations in the cortex.

It is challenging to separate the impact of local recurrent activity from feedforward and feedback activity as in most physiological settings all three mechanisms occur simultaneously. Previous work has focused on isolating recurrent activity by removing either feedforward or feedback activity. Several studies measured feedforward thalamic inputs in isolation by recording the responses to visual stimuli while reversibly silencing the cortex^4,11–14^, while other studies^15,16^ silenced higher brain areas through cooling to remove feedback signals.

In our study, we take a complementary approach—we use high-resolution two-photon (2P) holographic optogenetics to recreate precise experimenter-controlled patterns of neuronal activity and simultaneously measure the impact across V1 using cellular resolution 2P calcium imaging^17,18^. With this strategy, we probe the functional logic of recurrent cortical dynamics in the absence of visual-driven input and unambiguously determine the causal impact of local recurrent dynamics. However, the space of 2P holographic optogenetic stimulation protocols is immense and care must be taken to parameterize a sufficiently rich, yet feasible, probe of the recurrent circuit.

Two main organizing principles governing recurrent wiring in layer (L) 2/3 of mouse V1 are as follows: first, excitatory (E) and inhibitory (I) connectivity falls off with the physical distance between neurons, such that most recurrent connectivity comes from neurons that are less than 200 μm apart^19–24^. Second, E-to-E connectivity is biased to occur between neurons with similar stimulus feature preferences, such as orientation tuning^3,5,21,22^. Many models of cortical circuits, both mathematical and conceptual, consider either spatial- or feature-dependent wiring. However, due to the large number of parameters, few consider how their interaction determines overall network response. Furthermore, while knowledge of monosynaptic connectivity is essential for predictive models of recurrent cortical dynamics, it is not sufficient. Both cortical nonlinearities and multisynaptic paths complicate the relationship between physical synaptic connections and their functional influence on network activity. We hypothesize that by designing our 2P optogenetic stimulation to probe recurrent circuitry defined by both physical space and feature preference we can uncover the rules by which recurrence promotes either the recruitment or suppression of cortical activity.

Recent work using targeted photostimulation, concurrent with visual stimulation, probed the functional ‘influence’ of putative single-neuron perturbation in V1 (ref. ^10^). This study found that cotuned neurons tended to suppress each other, contrary to the prediction from the enriched ‘like-to-like’ connectivity between excitatory neurons^3^. In contrast, another study^25^ that stimulated larger ensembles of cotuned neurons found like-to-like activation. However, neither study focused on how the spatial arrangement and cotuning of the activated ensemble might jointly determine the net effect on the network. Because all activity patterns vary simultaneously across these two dimensions, a key aspect of recurrent dynamics in the cortex remains unexplored.

Previous rate-based modeling work showed that to reproduce such strong like-to-like suppression, the network must have strong and specific E-to-I connections^26^. Meanwhile, another computational study predicted that adjusting the stimulus contrast would shift the network and yield like-to-like activation^27^. These results highlight the importance of measuring and modeling functional interactions in the intact circuit.

The influence of a single neuron can be quite different from that of an ensemble of neurons with coordinated activity. Multicell photostimulation has revealed diverse functional interactions in L2/3 (refs. ^25,28–30^). Consequently, creating generalized organizing principles for the impact and function of L2/3 recurrent circuits remains difficult.

To define the functional logic of recurrent cortical dynamics in L2/3 of V1, we precisely photostimulated ensembles of excitatory neurons with 2P holographic optogenetics. We photoactivated ensembles of cells organized along the following two fundamental axes of the visual representation: physical space and feature space (orientation). Taken together, our data reveal two key organizing principles that eluded prior investigation that only probed along one of these axes at a time. While most perturbations generate net suppression, many ensembles drive amplification specifically in nearby (<30 μm) cells. This amplification is strongest when diffuse untuned ensembles are stimulated; however, when a cotuned ensemble is driven, it will primarily affect similarly cotuned neighbors. Conversely, compact cotuned ensembles generate the strongest suppression, leading to net suppression at all distances. A linear rate-based computational model captured these key results, but only if we incorporated a wiring rule that combines spatial- and feature-base synaptic organization. Specifically, we find that the model requires highly local like-to-like excitatory connections and the convergence of cotuned excitatory neurons onto local cotuned inhibitory neurons. This combination of all-optical circuit interrogation and detailed computational modeling demonstrates that neural representations in feature space and physical space intimately interact in V1. Furthermore, they outline organizing principles for the functional impact of recurrent cortical dynamics, distinguishing the conditions for when feedback amplifies input versus when it drives competitive suppression.

## Results

**Extended Data Fig. 1:**
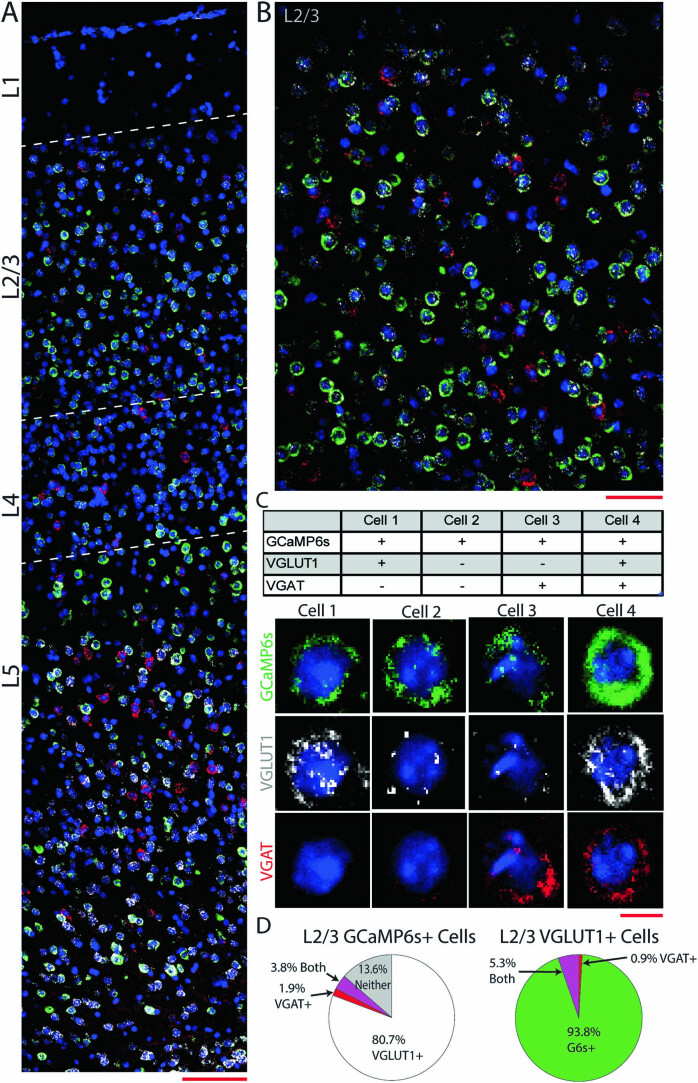
RNAscope validation of cell type specificity. **a:** A cortical section of a tetO-GCaMP6s x Camk2a-tTA mouse using RNAscope. GCaMP6s (green), VGLUT1 (white), VGAT (red), and DAPI (Blue) are labeled. Approximate division of cortical layers noted by dashed lines. Scale bar 100 µm. Representative image from 5 similar cortical slices. **b:** Enlarged view of L2/3 cortex. Scale bar 50 µm. **a,b:** Representative images from 5 coronal slices from one animal. **c:** Example L2/3 cells with expression of GCaMP6s, VGLUT1, and/or VGAT noted. Scale Bar 5 µm. **d:** Quantification of N = 300 DAPI positive cells from 5 slices, presented as either GCcaMP6s+ cells that co-stained for markers of VGLUT1, VGAT, or both (left) or VGLUT1+ cells that co-stain for GCaMP6s, VGAT, or both (right). 84.5% of GCaMP6s+ cells stain for VGLUT1, 99.1% of VLGUT1+ cells stain for GCaMP6s.

To determine the role of recurrent activity in L2/3, we used holographic 2P optogenetics to drive small ensembles of L2/3 cells in the absence of visual stimuli, thereby isolating their local network impact. We used three-dimensional scanless holographic optogenetics with temporal focusing (3D-SHOT)^17,18^ and leveraged potent ultrafast opsins^18,31^ that together enable the activation of dozens of cells with near single-cell resolution and millisecond precision. We simultaneously read out the activity of both stimulated and unstimulated cells using GCaMP6s. We restricted both the calcium sensor and the opsin to excitatory neurons (Methods; Extended Data Fig. 1) and imaged and photostimulated in 3D to obtain read/write control over a large fraction of the L2/3 V1 excitatory network. We first tested each opsin-expressing neuron for photosensitivity and tailored the laser power for each cell to ensure reliable activation (Methods). Next, we imaged responses of this population to orientated drifting gratings, determining each neuron’s orientation tuning online. Finally, we constructed ensembles of neurons with varied distributions of net orientation tuning and spatial locations and targeted these cells for 2P holographic photostimulation (for detailed cell selection criteria, see Methods).

### Heterogenous suppression dominates recurrent network effects

**Fig. 1:**
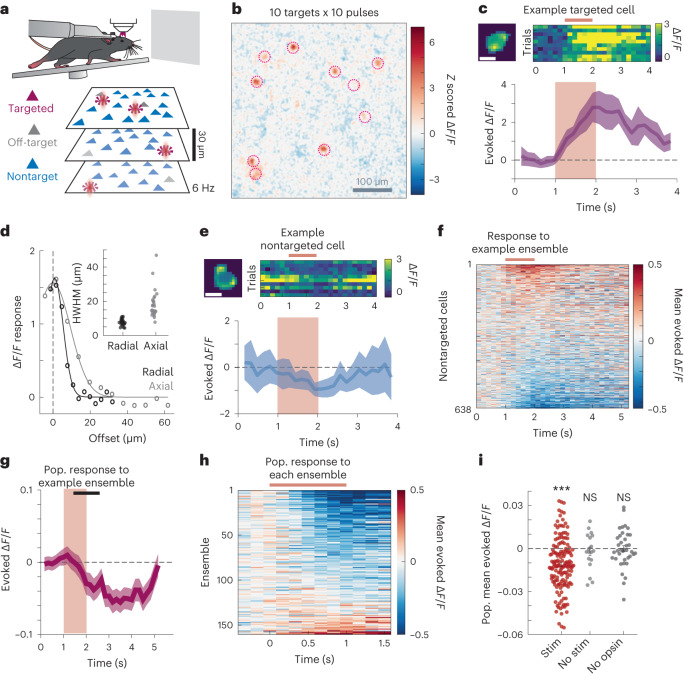
Stimulation of ten-cell ensembles recruits net suppression. **a**, Schematic representation of the experimental setup. Head-fixed mice run on a treadmill in front of a gray screen. Cells from three different planes are imaged at 6 Hz. Cells from any plane can be targeted for photostimulation (magenta), cells adjacent to photostimulated cells (including offset axially) are categorized as ‘off-target’ and excluded (gray), while remaining detected cells are ‘nontarget’ cells and used for analysis (blue). **b**, Image of the average modulation across three superimposed planes of imaging during stimulation of a representative ten-cell ensemble. Image pixels are *z* scored over the entire recording and averaged by trial type. **c**, Response of a targeted cell to photostimulation. Top left, image of the cell mask used in analyses, scale bar 10 µm. Top right, response of this neuron during ten photostimulation trials, stimulation time noted by the maroon bar. Bottom, average evoked calcium response from the same example cell. Mean evoked Δ*F*/*F* ± 95% confidence interval (CI). Maroon box denotes the stimulation time (ten pulses at 10 Hz). **d**, Representative PPSF aligned to peak (dotted line). Inset PPSFs from 25 cells throughout the FOV. **e**, Response of an example nontarget cell to ensemble stimulation, as in **c**. **f**, Mean response of 638 nontarget cells from a single FOV in response to a representative ensemble stimulation. Cells are sorted based on their response magnitude. Stimulation time noted by a maroon bar. **g**, Mean ± 95% CI of all 638 nontarget cells from **f**. Maroon box indicates stimulation time, and the black bar indicates the analysis window. **h**, Population response, that is, mean response of all nontarget cells, in a FOV to 160 unique ten-cell ensembles. *n* = 160 ensembles, 18 FOVs, 13 mice. **i**, Mean population response for each ensemble stimulation (maroon, *n* = 160 ensembles, 18 FOVs, 13 mice), no stimulation controls (gray, *n* = 18 FOVs, 13 mice), or no opsin controls (black, *n* = 38 ensembles, 1 FOV, 1 mouse). Mean ± s.e.m. of condition in black. Triple asterisks indicate a significant difference from 0, and NS indicates not significant (stim, *P* = 1.7 × 10^−8^; no stim, *P* = 0.79; no opsin, *P* = 0.96; two-sided signed rank test).

**Extended Data Fig. 2:**
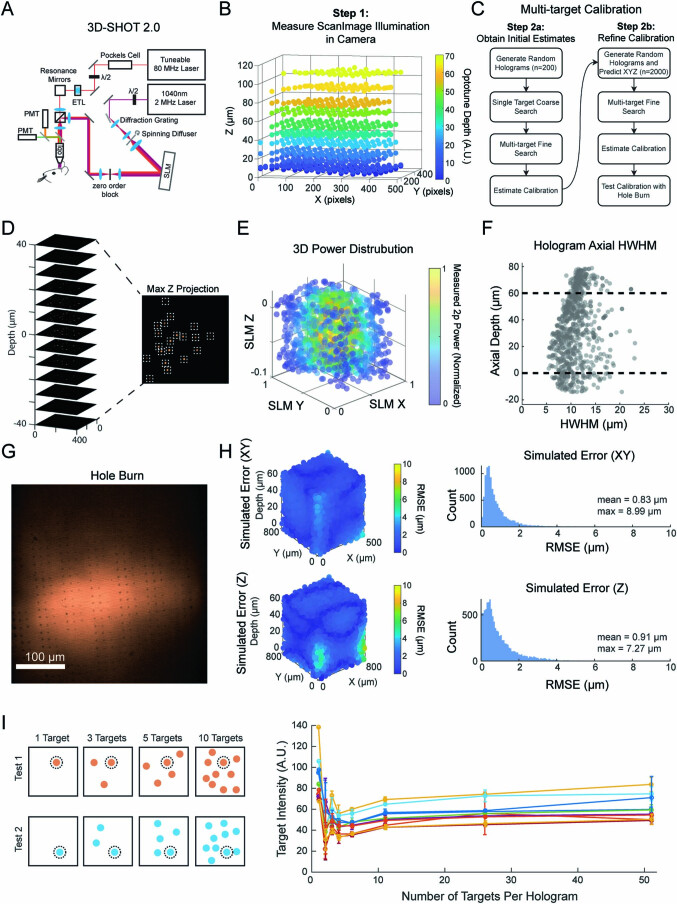
3D-SHOT2.0 and 3D spatial calibration. **a:** Microscope setup implementing simultaneous holographic optogenetics (via 3D-SHOT 2.0) and 2-photon calcium imaging. Imaging and stimulation paths are co-aligned and calibrated using the procedure outlined in this figure. **b:** First, a substage camera is used to image the ‘imaging’ fields of view in three dimensions. A thin fluorescent slide is imaged by the camera, and the microscope focus moved above and below the slide to gain depth information. The field curvature, introduced by the ETL, among other elements, was measured as a function of XY position and depth and then fit with a polynomial. Multiple ETL offsets representing the different imaging planes that might be used in an experiment are imaged separately. **c:** Second, we devised a multiplexed approach to register the position and diffraction efficiency of hologram targets (aka individual illuminated spots) throughout the Imaging FOV. To begin, the approximate XYZ position of 200 randomly selected single-target holograms was determined by imaging a with the substage camera. Each hologram was projected onto the thin film slide, and then a z-stack of images made by defocusing the microscope. The XY position of each hologram was determined by the position on the camera, while the Z position inferred from the stack. Next, multi-target holograms comprised of the holograms imaged in the initial phase were imaged at a higher resolution to provide a more accurate XYZ position. By imaging holograms as multi-targets we were able to parallelize data acquisition and dramatically improve calibration times. Next, we use the initial 200 imaged holograms to estimate the calibration and registration of holograms in the imaging FOV. Using these initial model fits, we then generate 100 20-target holograms (for a total of 2000 data points), predict their location in the substage camera, and extract the resulting XYZ position, relative power, and HWHM throughout the entire imaging FOV. Data were fit with a polynomial to generate a general transform for any SLM and imaging coordinate. Finally, we test the spatial calibration with an automated ‘hole-burn’ (n = 300 holes burnt during calibration). **d**. An example of a multi-target hologram being imaged in a stack. **e:** Example power distributions in 3D (presented in arbitrary SLM units). Power distributions vary significantly throughout 3D space. By modeling hologram diffraction efficiency in 3D, we can dynamically compensate power during an experiment to accurately use a greater range of the SLM. Each point represents a single imaged hologram; color represents the relative power from that hologram. **f:** Axial hologram HWHM varies as a function of depth. We co-aligned the axial position of the imaging and holographic stimulation pathways such that the holograms with smallest HWHM were positioned within the typical Imaging axial range used in experiments (dashed lines). Each point represents a single imaged hologram. **g:** Example image from the automated ‘hole-burn’. A unique pattern is bleached/burned onto a thin fluorescent slide, imaged in ScanImage, and the XY position of hole-burn locations is detected. This process is repeated for multiple z-planes. A ‘hole-burn’ image is taken for each hole, consequently this image is one of ∼300 images per calibration, and representative of the burns made in all >5 Calibrations performed over this project. **h:** With the calibration in place, we simulated XY (top) and Z (bottom) error in targeting throughout the typical imaging field of view (n = 10,000 simulations). Root-mean-square error (RMSE, in µm) is presented in 3D (left) and overall error distributions (right). **i:** To understand the variability in delivered power across different types of holograms, we measured the fluorescence evoked by a series of 10 test targets (dotted circle, left). Using the substage camera, we measured the intensity from the test target alone in a single-target hologram, or in a hologram that also contained 1 to 50 randomly chosen ‘distractor’ targets, 10 repeats with new distractor targets were performed per test target and hologram size. *Right*, the intensity of each test target as a function of the total number of targets in the hologram. Each color denotes a different test target, while the variability comes from different ‘distractor’ targets (presented as S.E.M).

**Extended Data Fig. 3:**
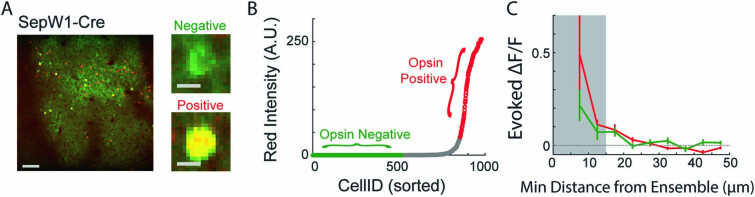
Sparsely expressing mice for analysis of opsin negative cells. **a**: Representative image from one of 6 FOVs from a SepW1-Cre mouse with AAV CAG-DIO-ChroME2. Scale Bar 100 µm. Inset, example images of opsin negative (above) and opsin positive (below) cell. Scale Bar (10 µm). Green is from GCaMP, red from nuclear localized mRuby3 part of the opsin construct. **b:** The intensity of the red fluorophore detected for each cell in one field of view (representative of all 6). Top 80^th^ percentile categorized as opsin positive, bottom 30^th^ percentile opsin negative (Note: red counts are integers and more than 30% of cells may have the same or lower 30^th^ percentile score). **c:** Evoked ΔF/F responses in non-targeted cells to 10 cell ensemble stimulation as a function of distance from the closest target, separated by opsin positive and opsin negative cells. Exclusion zone (15 µm) marked in gray. N = 57 ensembles, 6 FOVs, 4 Mice.

We first asked how activating a small number of opsin-expressing L2/3 excitatory cells (groups of ten targeted cells) would impact overall activity in L2/3 (Fig. 1a,b). Strong activation of the targeted cells confirmed the efficacy of the optogenetic approach (Fig. 1c). However, in all optogenetic experiments, stray light can inadvertently activate nontargeted cells. To exclude such cells from analysis, we developed an extensive 3D calibration (Methods; Extended Data Fig. 2) resulting in a high-quality optical point-spread function (PSF) and a physiological PSF (PPSF) across the entire targeting volume. We use this PPSF to define our off-target zone in which all neurons were excluded from analysis (Methods, mean PPSF half-width half maximum (HWHM) radial 7.73 ± 0.37 µm, axial 18.51 ± 1.69 µm, *n* = 25; Fig. 1d). Next, we executed an additional set of experiments in mice with sparse opsin expression (SepW1-Cre;Camk2a-tTA; tetO-GCaMP6s mice) and found that opsin-negative and opsin-positive cells outside of this exclusion zone responded identically (Extended Data Fig. 3), indicating that effects beyond this spatial threshold are not due to off-target light.

**Extended Data Fig. 4:**
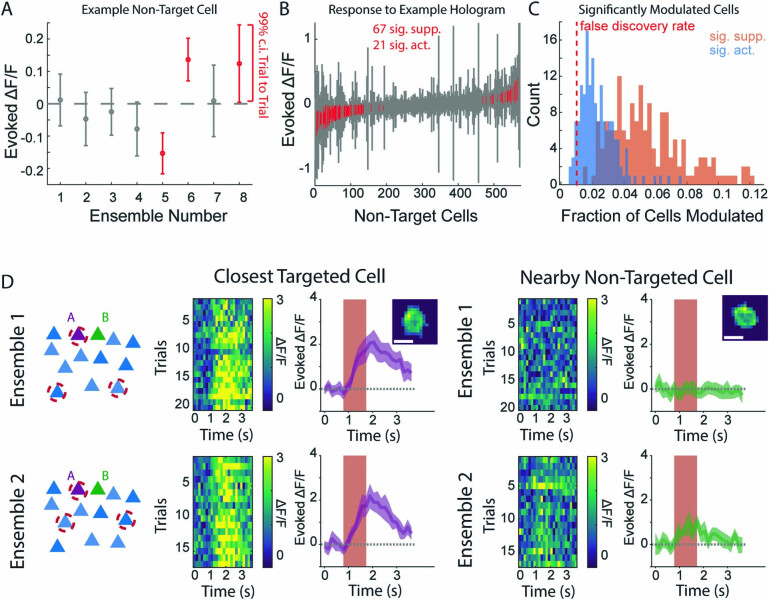
Heterogeneity in responses to holographic stimulation. **a:** Evoked ΔF/F responses in a single example non-target cell to 8 distinct 10 cell ensembles stimulations from the same recording. Trial variability presented as mean ± 99% c.i. Significantly modulated conditions (that is, 99% c.i. excludes 0) are marked in red. Each ensemble was stimulated in at least 10 trials (see Methods) **b:** All non-target cells from a given field of view response to a single example hologram. Mean ± 99% c.i. sorted by mean response. Cells that are significantly modulated (that is, 99% c.i. excludes 0) marked in red. To this hologram 67 cells were significantly suppressed, while 21 were activated. **c:** For each stimulated ensemble, the fraction of non-target cells significantly activated (blue) or suppressed (orange) (via 99% c.i.) is presented as a histogram. False discovery rate (1%) is noted by the dotted red line. N = 160 Ensembles, 18 FOVs, 13 Mice. **d:** An example pair of cells that were close to each other (<30 µm) but responded differently to different, but similar, ensembles. Cell A (purple) was targeted in both Ensemble 1 (top) and Ensemble 2 (bottom). Cell B (green) was never directly targeted but was silent in ensemble 1 and driven in ensemble 2. Each row shows a schematic of the ensemble (far left), color plot of the fluorescence observed each trial for cell A (left), mean ± 95% c.i. response for cell A (middle), color plot of the fluorescence observed each trial for cell B (right), and mean ± 95% c.i. response for cell B (far right). Ensemble 1 was repeated in 20 trials, ensemble 2 in 17 trials.

**Extended Data Fig. 5:**
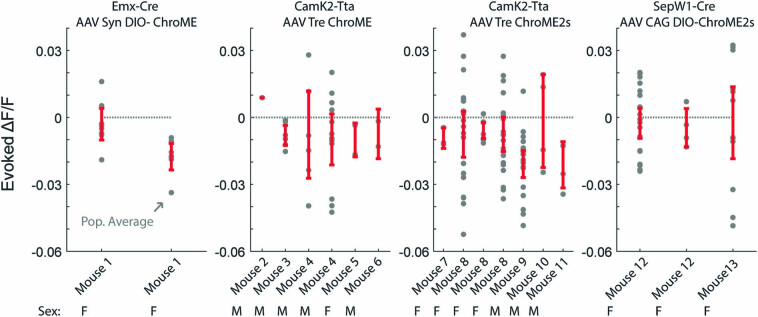
Comparison of effects by mouse and preparation. Mean population average of all non-targeted cells in response to each 10 cell ensemble stimulation (gray dot). Average ± SEM for each recording FOV in red. Divided by expression selectivity driver (Emx-cre, CamK2-tTA, or SepW1-Cre) and viral construct (Syn-DIO-ChroME, Tre-ChroME, Tre-ChroME2s, or CAG DIO-ChroME2s). Sex of each mouse is noted below. Note SepW1-Cre ensembles are more likely to be spread out, due to the sparse nature. Each dot is the mean response to an ensemble across all conditions N = 160 Ensembles, 18 FOVs, 13 mice.

Nontargeted neurons displayed varied effects, with individual cells responding distinctly to different ensembles (Extended Data Fig. 4). However, most cells were suppressed in response to ensemble stimulation (Fig. 1e–g and Extended Data Fig. 4b,c). Across a large set of experiments (160 unique ten-cell ensembles in 18 fields of views (FOVs) in 13 different mice), we found that photostimulation suppressed mean population activity (mean effect: −0.011 ± 0.0014 Δ*F*/*F*, *P* = 1.0 × 10^−10^, Wilcoxon two-sided rank sum test; Fig. 1h,i). Responses of individual FOVs were similar across mice and preparation (Extended Data Fig. 5). We only included trials in our analyses where we could confirm that most targeted cells were activated, and each included ensemble had at least 11 repetitions (median 17; range 11–32 trials). These results indicate that photostimulation of a small number of L2/3 excitatory neurons recruits net suppression across the entire population at least in the absence of a visual stimulus.

**Extended Data Fig. 6:**
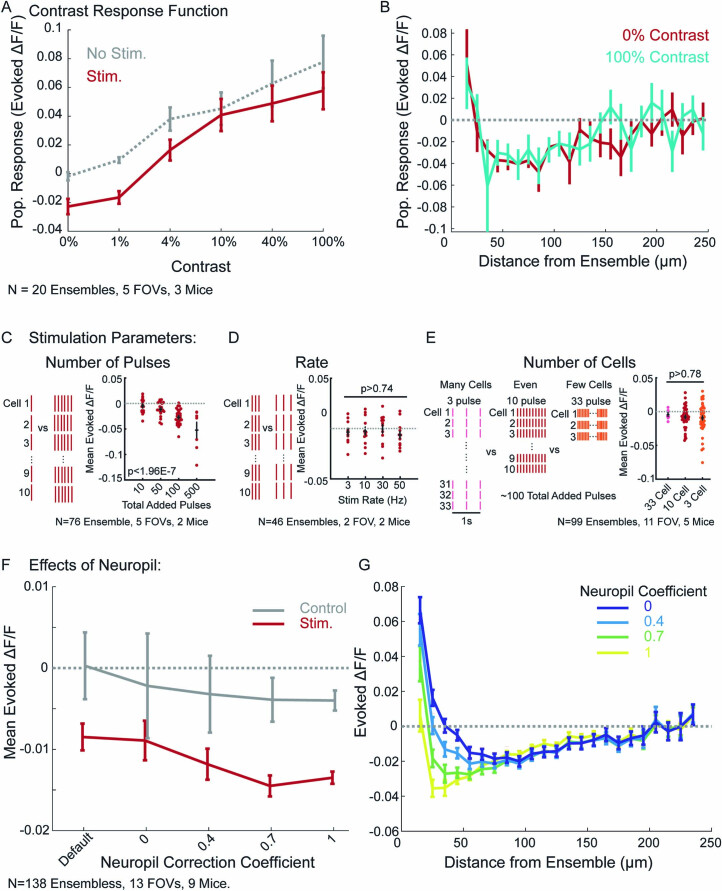
Determining experimental conditions. **a**,**b** Ensemble activation during visual stimulus. **a**: average population response of all (non-targeted) recorded neurons to visual stimulus alone (Gaussian modulated noise of various contrasts) and with concurrent photo-stimulation of 10 neurons with 10 photo-stimulation pulses. **b**: Mean population response of all non-targeted neurons as a function of their distance from the photo-stimulated ensemble across 0% and 100% contrast. The mean visual response has been subtracted off to facilitate comparison. All other figures are in the absence of visual stimuli. N = 20 Ensembles, 5 FOVs, 3 Mice. **c–e** Observed decrease in fluorescence is dependent on the number of spikes added. **c:** In the absence of visual stimuli, number of total pulses added increased overall mean suppression. Mean evoked population response to 10 cell ensemble stimulation, with 1, 5, 10 or 50 pulses per cell. P = 1.97e-7 1-way ANOVA, N = 76 Ensembles, 5 FOVs, 2 Mice. **d:** Rate of stimulation did not affect the overall mean suppression. Mean evoked population response to 10 cell ensemble driven with 10 pulses at 3, 10, 30 or 50 Hz. P = 0.74 1-way ANOVA, N = 46 Ensembles 2 FOVs, 2 Mice. **e:** Number of cells stimulated, when holding number of spikes constant, did not change overall suppression. Ensembles of 33 cells driven with 3 pulses, vs 10 cells driven with 10 pulses, vs 3 cells driven with 33 pulses did not recruit a differential amount of suppression. P = 0.78 1-way ANOVA, N = 99 Ensembles, 11 FOVs, 5 Mice**. f**,**g** Neuropil correction does not explain suppression. **f**: Overall average response of non-targeted neurons (n = 138 ensembles, 13 FOVs, 9 Mice), calculated with a variety of neuropil subtraction coefficients. **g**: Response of non-targeted neurons as a function of distance from stimulated ensemble members (as in Fig. 2a) calculated with a variety of neuropil subtraction coefficients (same ensembles as panel F). All data presented as mean ± SEM.

Separately, we paired optogenetic stimulation with visual stimulation (Gaussian modulated noise of varying contrast), but the overall suppressive effect was similar (Extended Data Fig. 6a,b). It is possible that different types of visual stimuli modulate these effects, but we chose to focus on recurrent dynamics in the absence of a visual stimulus to isolate it from external input.

As different patterns of activity may recruit distinct circuits that preferentially drive either activation or suppression^10,25^, we next asked whether the overall sign and magnitude of recurrent activity during photostimulation depended on how the pulses were added to the system. First, we varied the total number of pulses delivered to a group of ten targeted neurons between 1 and 50 pulses per cell (∼10 to 500 total pulses), while holding the pulse frequency and ensemble size constant (10 Hz and 10 cells, respectively). We observed net suppression across all these conditions, with a monotonic increase in suppression with increased number of spikes (*P* = 1.7 × 10^−8^, analysis of variance (ANOVA), *n* = 76 ensembles, 5 FOVs, 2 mice; Extended Data Fig. 6c). Next, we varied the stimulation frequency while holding ensemble size and total added pulse number constant. In contrast, varying the rate of stimulation did not change the magnitude of the net mean suppression (*P* = 0.74, ANOVA, *n* = 46 ensembles, 2 FOVs, 2 mice; Extended Data Fig. 6d). Likewise, varying ensemble size while adding a fixed total number of spikes also drove similar net suppression (mean Δ*F*/*F*: −0.0045 ± 0.0027 (3 pulses in 33 cells), −0.0081 ± 0.0023 (10 pulses in 10 cells), −0.0094 ± 0.0031 (33 pulses in 3 cells), *P* = 0.78, ANOVA, *n* = 99 ensembles, 11 FOVs, 5 mice; Extended Data Fig. 6e). Smaller ensembles drove more variable population responses (s.d. Δ*F*/*F*: 0.007 (33 cell ensembles) versus 0.015 (10 cell ensemble) versus 0.021 (3 cell ensemble), *P* = 0.006 Bartlett variance test). These results demonstrate that the primary driver of network suppression is the total number of added spikes, not the frequency of stimulation or the size of the ensemble.

Based on these results, we focused on the effects of adding 100 total pulses to ten targeted neurons, which represents a modest perturbation to the system that still drove reliable and readily quantifiable effects. Importantly, such modest perturbations are well captured through simulations of associated network models (see below) because they can be modeled as a linear perturbation around the network’s steady state.

While it is true that ensemble stimulation leads to suppression on average across the population, there remains significant heterogeneity in responses, with a significant number of neurons showing activation rather than suppression. After excluding potential off-targets, we found that 2.34 ± 0.09% of nontargeted cells were significantly activated (that is, 99% CI excludes 0, false discovery rate 1%), while 5.58 ± 0.19% of nontargeted cells were significantly suppressed (Extended Data Fig. 4b,c). The central goal of this study is to explain this heterogeneity of neural modulation based on the joint physical and feature space properties of the neurons both in the stimulated ensemble and recorded populations.

### Cortical space organizes the impact of recurrent dynamics

**Fig. 2:**
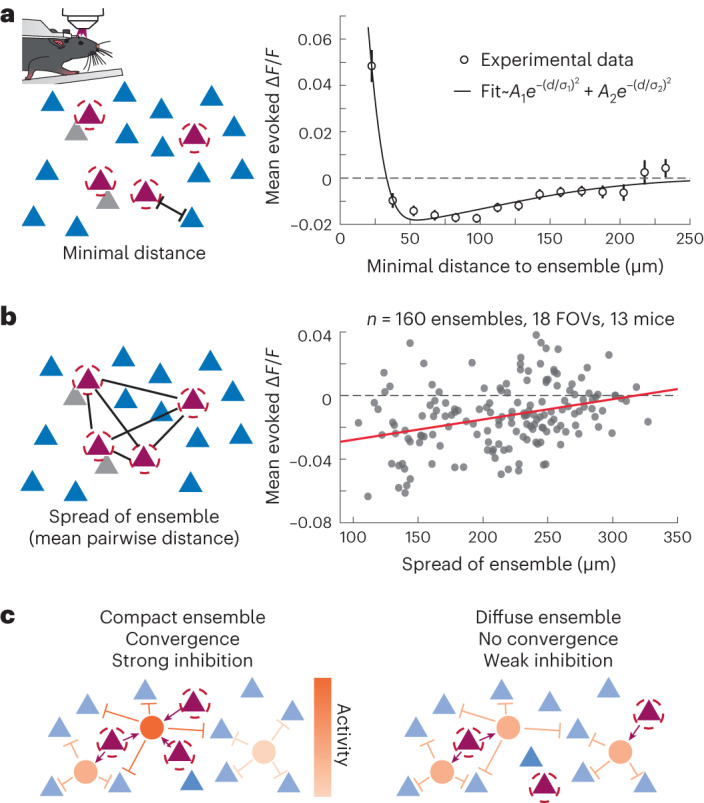
Population responses reveal nearby activation and surround suppression, with total recruited suppression dependent on ensemble spread. **a**, Left, schematic representation of the minimal distance metric for nontarget cells (magenta, targeted cells; gray, off-target cells and blue, nontargeted cells). Right, nontargeted cell responses to optogenetic stimulation as a function of minimal distance to ensemble (open circles) fitted to a sum of Gaussian spatial functions (*A*_1_ = 0.196, *σ*_1_ = 22.1 µm, *A*_2_ = −0.021, *σ*_2_ = 147.3 µm). Cells near the stimulated ensemble were reliably activated, while cells further away from a target were suppressed (<30 µm from a target mean Δ*F*/*F*: 0.044 ± 0.005, *P* = 1.1 × 10^−10^; 50–150 µm from a target mean Δ*F*/*F*: −0.013 ± 0.001, *P* = 4.0 × 10^−17^, two-sided signed rank test). Bin sizes are 15 µm. Mean ± s.e.m. **b**, Left, schematic representation of ensemble spread metric (mean pairwise distance). Right, nontargeted cell responses averaged across the population (*n* = 160) for ensembles with different spreads. Activating a spatially compact ensemble drives more surround suppression than stimulating a spatially diffuse ensemble (linear regression of mean Δ*F*/*F* versus spread slope: 1.3 × 10^−4^ Δ*F*/*F* per µm spread, *P* = 1.4 × 10^−5^). **c**, Schematics illustrating the ability of the targeted cells to activate inhibitory pathways via convergences for compact (left) and diffuse (right) ensembles.

Physical space in the sensory neocortex represents a fundamental axis of circuit organization owing to both the topographic mapping of sensory inputs onto cortical tissue and the anatomy of cortical neurons^32–35^. Thus, we hypothesized that the sign, scale and magnitude of recurrent circuit influence might vary substantially with distance from the targeted ensemble. To test this, we quantified the impact of ensemble photostimulation as a function of distance from each targeted location and ensemble (Methods). Indeed, we found that despite the overall mean suppression described above, cells proximal to the stimulated ensemble but outside of the off-target exclusion zone were reliably activated, while cells further away from a target were suppressed (<30 µm from a target mean Δ*F*/*F*: 0.044 ± 0.005, *P* = 1.1 × 10^−10^; 50–150 µm from a target mean Δ*F*/*F*: −0.013 ± 0.001, *P* = 4.0 × 10^−17^, two-sided signed rank test; Fig. 2a). Beyond that distance, the sign of the modulation stayed negative and slowly returned to zero as the distance increased. This general pattern was not affected by the presence of visual input (Extended Data Fig. 6b).

While direct photoactivation of adjacent cells is a risk, we do not believe this accounts for the observed nearby activation. We set our exclusion criteria conservatively based on our hologram PSF (Extended Data Fig. 2f) and measured PPSF (Fig. 1d; Methods). We confirmed that nearby opsin-negative cells behave the same as opsin-positive cells (Extended Data Fig. 3). Individual cells could be activated or suppressed to different holograms (Extended Data Fig. 4a) even when the nearest targeted cell is the same (Extended Data Fig. 4d). Furthermore, we recalculated our results using various neuropil subtraction coefficients (0–1) to account for potential signal contamination from activated cells on nearby nontargeted cells. Activation nearby was consistently observed (Methods; Extended Data Fig. 6f,g).

This spatial pattern of nearby activation and surround suppression characterizes the spatial response function of a minimal recurrent circuit and has often been considered as a basis for lateral competition in the cortex^9,36–40^. Phenomenologically, it can be captured as the difference of a narrow excitatory and a broader inhibitory Gaussian spatial function (Fig. 2a; solid line; excitatory spread: 22 μm, inhibitory spread: 147 μm). Moreover, these experiments demonstrate that activation of even a small number of L2/3 excitatory neurons is sufficient to generate this spatial pattern of modulation.

**Extended Data Fig. 7:**
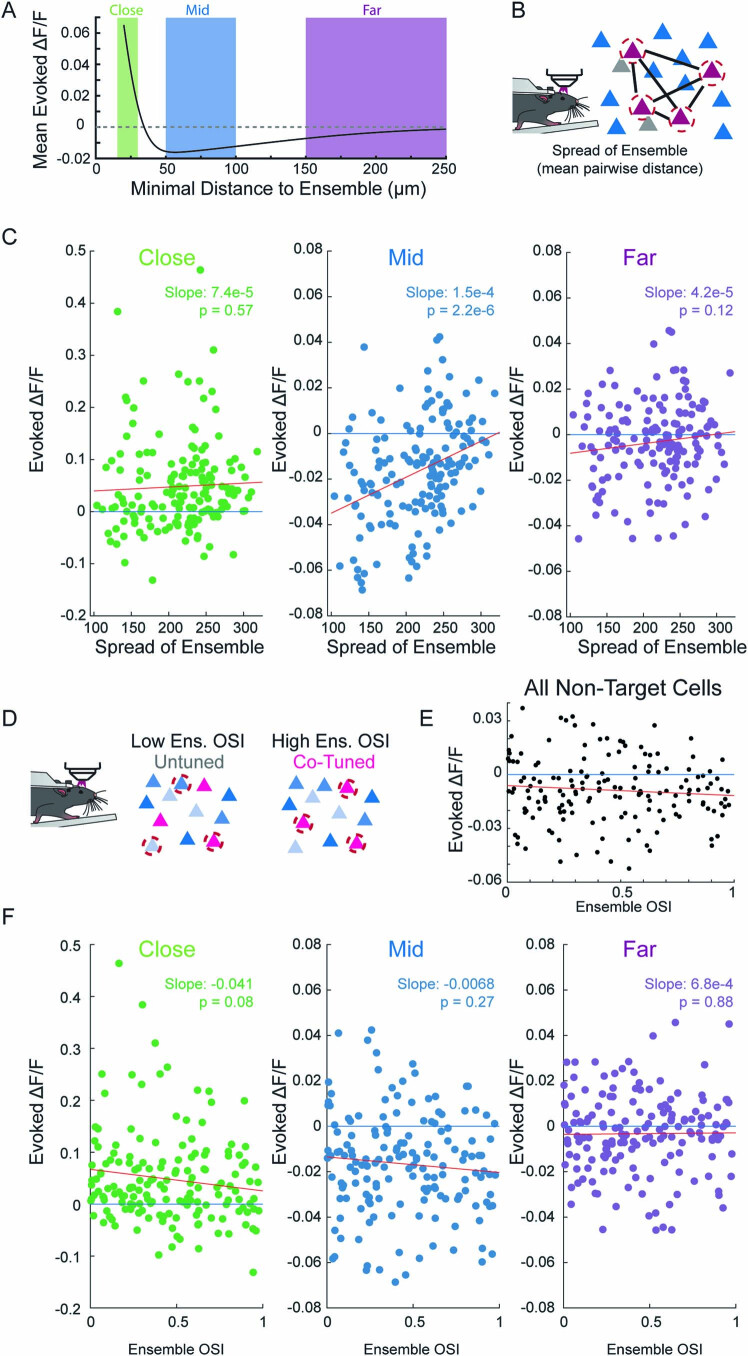
Correlation of population responses to ensembles statistics. **a:** Non-target cells are categorized based on their proximity to a targeted cell as close (<30 µm, green), middle (50–100 µm, blue) or far (>150 µm, purple) cells. These categories are plotted against the predicted average response from Fig. 2a. **b:** Schematic showing the mean pairwise distance of a stimulated ensemble. **c:** Population averages of evoked fluorescence from non-target cells categorized as close (green), middle (blue), or far (purple) from a targeted cell as a function of the ensemble spread. Each dot is a population response to an ensemble, N = 160 Ensembles, 18 FOVs, 13 mice. Blue line 0 effect, red line linear regression fit. Slope and p value for the *F*-test of the linear regression model written on the plot. **d:** Schematic showing cotuned vs untuned ensembles. **e:** The evoked fluorescence from all non-targeted cells regardless of distance to a stimulated cell as a function of ensemble OSI (that is, tuning). **f:** As in **c**, but as a function of ensemble OSI.

Like how different sensory stimuli drive different spatial distributions of activity, we next asked how the spatial distribution of targeted cells would impact either the suppression or activation of recurrent activity. To investigate this question experimentally, we activated ensembles of ten neurons that varied in how they were distributed in space (Fig. 2b). Indeed, we found that activating a spatially compact ensemble drives much more surround suppression than stimulating a spatially diffuse ensemble (linear regression of mean Δ*F*/*F* versus spread slope: 1.3 × 10^−4^ Δ*F*/*F* per µm spread, *P* = 1.4 × 10^−5^; Fig. 2b). In contrast, the spread of the ensemble did not alter nearby activation (linear regression of mean Δ*F*/*F* versus spread slope: 3.5 × 10^−5^, *P* = 0.57; Extended Data Fig. 7a–c). More precisely, the level of surround suppression (at 50–150 μm) increased as the spatial distribution of the ensemble’s component neurons decreased. We hypothesize that this strong inhibition derives from the convergence of excitatory activity onto individual inhibitory neurons. In the case of a spatially compact ensemble, individual inhibitory neurons receive input from multiple directly stimulated cells. These super-activated inhibitory cells then feedback divergently inhibiting the entire network (Fig. 2c).

### Feature space organizes the impact of recurrent dynamics

In addition to physical space, feature space represents a second axis of the functional organization of cortical circuits. In mouse V1, orientation tuning is a crucial feature that is not structured in physical space, unlike the orientation columns of monkeys, cats and other species^41–43^. Despite the lack of local organization, feature space is known to influence both synaptic connectivity and the functional influence of individual neurons in mouse V1 (refs. ^3,5,10^). However, as most reports focus on the impact of individual neurons, it is unknown if multiple neurons defined in feature space synergize to drive recurrent activity. One hypothesis is that a cotuned (that is, similarly tuned or iso-oriented) group of excitatory neurons, analogous to spatial clustering in orientation space, should drive strong network activity due to convergent excitation onto the same postsynaptic excitatory cells^25^.

**Fig. 3:**
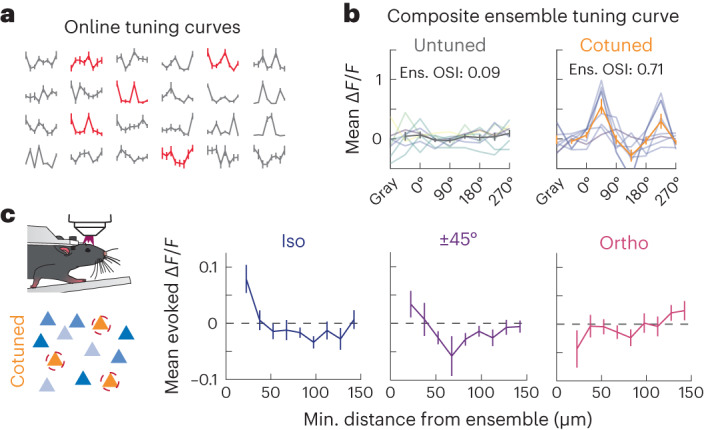
The relative match of orientation tuning of nontargeted cells to the tuning of a stimulated ensemble determines their modulation. **a**, Representative tuning curves (computed online) from 24 nontargeted cells. Each point of each curve is the mean ± s.e.m. normalized calcium response to a drifting grating. Red tuning curves are for cells that were selected to compose an individual ensemble. **b**, Two representative ensemble tuning curves. Left, a representative untuned ensemble (*n* = 8 cells). Right, a representative cotuned ensemble (*n* = 7 cells). Individual tuning curves lighter colors, mean ± s.e.m. dark bold colors. All ensembles are composed of a mix of visually responsive, tuned and unresponsive cells to create an ‘ensemble’ matching its desired features. **c**, Left, schematic representation showing photostimulation of a cotuned ensemble. Right, nontargeted cell responses ±s.e.m. as a function of their minimal distance to the targeted ensemble, split according to their relative tuning with respect to the ensemble tuning of the targeted ensemble (left, Δ*θ* = 0° (iso); middle, Δ*θ* = ±45° and right, Δ*θ* = 90° (ortho)). Included cells were responsive to visual stimuli (*P* < 0.05) and tuned (OSI > 0.25). Nearby iso-oriented cells (<30 µm) are activated more than those cells that prefer orthogonal stimuli (*P* = 0.0035, Wilcoxon one-sided ranked-sum test, *n* = 17 ensembles, 8 FOVs, 3 mice). Bin sizes are 15 µm.

To test this hypothesis, we presented mice with a randomized series of full-screen drifting gratings each trial presenting one of eight cardinal directions of motion and calculated tuning curves for each neuron online, generating ensembles of cells varying in preferred orientation (PO) and orientation selectivity. We summarized the selectivity of an ensemble using an ‘ensemble OSI,’ that is, the orientation selectivity index (OSI) of the average of the tuning curves (Methods; Fig. 3a,b). To optimally select exemplar ensembles (among the ∼10^23^ possible ensembles—1,000 choose 10), we created a discrete optimizer (Methods) that designs distinct ensembles by automatically choosing eligible cells that fall within a targeted spatial and OSI range. We used this optimizer to ensure that the chosen ensembles were evenly distributed across feature (orientation) space. All ensembles are composed of a mix of visually responsive, tuned and unresponsive cells to create an ‘ensemble’ matching its desired features. Surprisingly, we found that ensembles with higher ensemble OSI did not generate greater network effects when averaged across all nontargeted neurons (Extended Data Fig. 7d–f; no significant correlation between ensemble OSI versus the total population response, *P* = 0.26 linear regression; or between ensemble OSI and nearby, mid-distance or far cells’ responses; *P* = 0.084, *P* = 0.27 and *P* = 0.89, respectively, by linear regression).

Based on the principle of selective like-to-like connectivity, we next asked whether cotuned ensembles might preferentially impact nontargeted neurons that share feature preferences. To test this hypothesis, we restricted our analysis to the ‘cotuned ensembles’ and divided the nonstimulated cells based on their relative PO. Such a ‘cotuned ensemble’ consisted of individually tuned members (mean OSIs of ensemble members >0.5) with similar tuning preferences (ensemble OSI > 0.7). We find that nearby cells (<30 µm) that also prefer the same orientation as the stimulated ensemble (that is, iso-oriented cells) are activated dramatically more than those cells that prefer orthogonal stimuli (*P* = 0.0035, Wilcoxon one-sided ranked-sum test, *n* = 17 ensembles, 8 FOVs, 3 mice; Fig. 3c). Strikingly, these nearby orthogonally oriented cells were instead highly suppressed. These results demonstrate that the organization of an ensemble in feature space—in this case, orientation preference—profoundly influences its recurrent impact on specific cells in the cortical network.

### Interactions between physical space and feature space

**Fig. 4:**
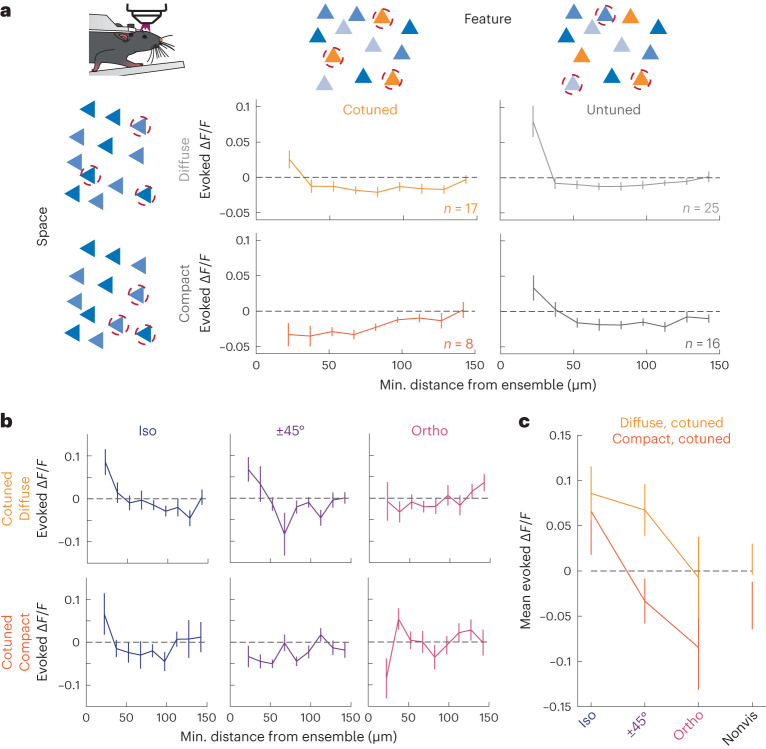
Space and feature properties of the photostimulated ensemble jointly control recurrent activity. **a**, Ensembles are divided into two categories as follows: in rows whether they are diffuse (mean distance >200 µm, top) or compact (<200 µm, bottom) versus in columns whether they are cotuned (Ens. OSI > 0.7, left) or untuned (<0.3, right). Schematics by the axes describe the ensemble design in that row/column. Data presented are mean nontargeted cell responses ± s.e.m. as a function of their minimal distance to the ensemble. Diffuse cotuned *n* = 17 ensembles, 9 FOVs, 3 mice; diffuse untuned *n* = 25 ensembles, 13 FOVs, 3 mice; compact cotuned *n* = 8 ensembles, 3 FOVs, 1 mouse; compact untuned *n* = 16 ensembles, 7 FOVs, 2 mice. **b**, Data from the left two panels of **a** now split by relative orientation tuning to the cotuned ensemble. Response plots of cotuned ensembles divided by mean separation (as in **a**), but nontargeted cells are separated by relative tuning with the stimulated ensemble (left, Δ*θ* = 0° (iso); middle, Δ*θ* = ±45° and right, Δ*θ* = 90° (ortho), *n* = 17 diffuse and cotuned ensembles, from 9 FOVs, 3 nice, *n* = 8 compact and cotuned ensembles, from 3 FOVs, 1 mouse). **c**, Similar to **b**, mean response of nontargeted cell responses during stimulation of diffuse, cotuned (*n* = 17) and compact, cotuned (*n* = 8) ensembles. Here data shown are from each of the first spatial bins of the six panels in **b** as a function of their relative tuning with the stimulated cotuned ensembles. All data are presented as mean ± s.e.m.

Thus far, we have only considered how the geometric distribution (Fig. 2) and feature preferences of an ensemble (Fig. 3) govern cortical recurrent dynamics independently. However, because sensory stimuli will necessarily recruit recurrent dynamics that vary jointly across these two dimensions, we hypothesized that the spatial distribution and feature preference of an ensemble should interact to determine the resultant impact of ensemble photostimulation on the cortical network. To investigate this, we used the discrete optimizer to identify cotuned or untuned ensembles that were either spatially compact or spatially distributed and photostimulated them while observing the activity of the nontarget cells. First, we found that the spatial spread of an untuned ensemble (ensemble OSI < 0.3 and mean OSIs of ensemble members <0.5; Methods) did not affect its net recurrent impact, such that for both spatially compact and diffuse ensembles we observed the characteristic nearby activation and surround suppression when computed across all nontargeted neurons (Fig. 4a, gray traces). However, the spatial spread of a cotuned ensemble profoundly influenced its recurrent effects—compact, cotuned ensembles generated no nearby activation and instead showed nearby suppression, whereas a spatially diffuse cotuned ensemble generated the more typical center/surround effects (Fig. 4a, light and dark green traces; nearby activity cotuned, close ensemble (*n* = 8) versus cotuned far ensemble (*n* = 17) *P* = 0.008, Wilcoxon one-sided ranked-sum test).

**Extended Data Fig. 8:**
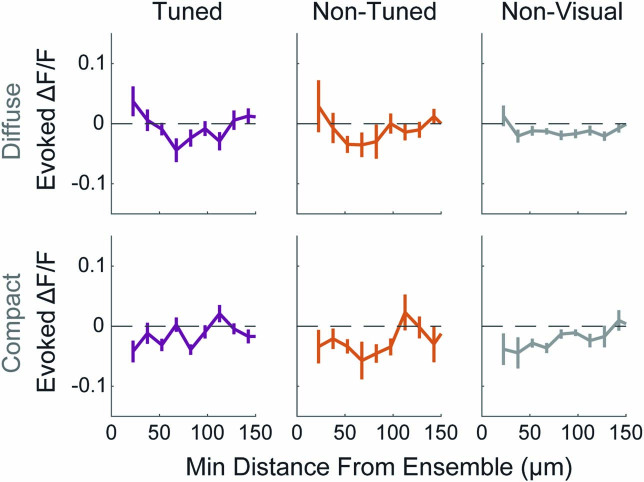
Responses of cells divided by their response properties. Response of non-targeted cells during cotuned ensemble stimulation, as in Fig. 4a*left*. All observed cells are categorized by their responses to oriented visual stimuli as either tuned, non-tuned, or non-visually responsive. N = 17 ensembles, 9 FOV, 3 mice. top row; N = 8 ensembles, 3 FOVs, 1 mouse bottom row. All data represented as mean ± SEM.

This result appears at odds with previous optogenetic results that have suggested that driving a cotuned ensemble should evoke a population response similar to that driven by a grating^25^. To reconcile these results, we asked how the activity of visually responsive cells varied across spatially diffuse and compact cotuned ensembles, as a function of their orientation preference (Fig. 4b,c). By subdividing the data from Fig. 4a by orientation preference, we observed that diffuse cotuned ensembles recruited little nearby suppression in neurons at any orientation. In contrast, compact cotuned ensembles activated nearby iso-oriented cells but suppressed nearby cells that prefer other orientations. Furthermore, both nonvisually responsive and untuned cells were also suppressed during the stimulation of such ensembles (Extended Data Fig. 8). All ensembles suppress the activity of further away cells, regardless of their tuning.

### Highly local excitatory connections drive nearby activation and surround suppression

**Fig. 5:**
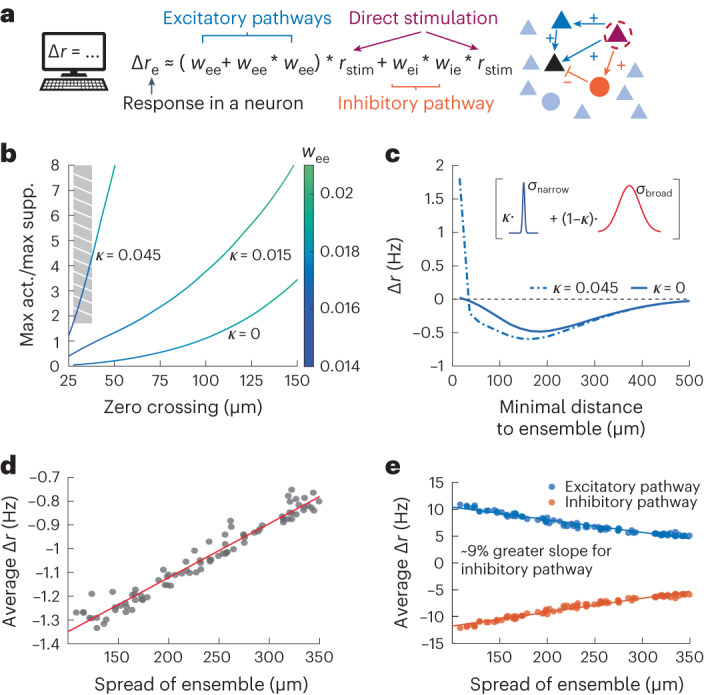
Mathematical model can capture nearby activity and surround suppression. **a**, Schematic representation of all monosynaptic and disynaptic pathways resulting in a change in the baseline firing rate (blue, excitatory pathways and orange, inhibitory pathways). An asterisk denotes convolution in space. **b**, The zero crossing and relative strength of nearby activation (maximum activation/maximum suppression) as a function of recurrent excitatory strength (*w*_ee_) and biased connections on the narrow spatial scale (*κ*). The gray stripe box indicates the experimentally observed data regime and illustrates the need for additional spatial constraints to capture the nearby activation observed in the data. **c**, Modulation of the activity of nontargeted cell responses in the model as a function of minimal distance to ensemble for different values of *κ*. Inset, schematic representation shows the narrow versus broad spatial scales in the model (see Methods for more details). **d**, Same as Fig. 2b except for the network model. **e**, Strength of the model excitatory (blue) and inhibitory (red) pathways as a function of ensemble spread, showing that as the ensemble spread decreases, the inhibitory pathway shows a greater level of recruitment.

**Extended Data Fig. 9:**
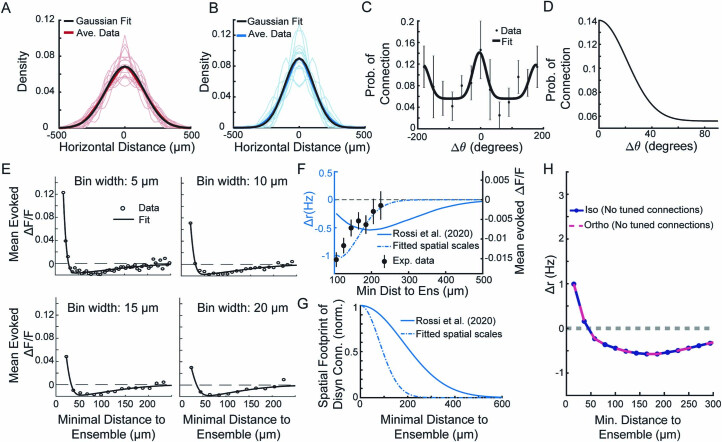
Using experimental data to fit spatial and feature spread parameters and the mathematical model with no tuned connections. **a:** Data from Rossi et al.^22^ used as a baseline for the broad spatial component of E → *α*, for *α* = E, I (σ_*e,b*_ = 147.31 µm). **b:** Same as **a**, except for I → *α* (σ_*i,b*_ = 111.27 µm). **c:** Data from Rossi et al.^22^ (n = 15 trials; data represented as median ± MAD) to for the feature-based connectivity rule for E → E connections (σ_ee_ = 21°, *r*_0_ = 0.056, *r*_*p*_ = 0.084). **d:** Zoomed in portion of panel **a** from 0° to 90°. **e:** Non-targeted cell responses to optogenetic stimulation as a function of minimal distance to ensemble for different (hollow circles) fitted to a sum of Gaussian spatial functions for different bin widths. The zero-crossing, maximum, and minimum values were estimated for each fitted function and used to determine the experimentally observed data regime box used in Fig. 2c. **f:** The spatial connectivity parameters suggested from Rossi et al.^22^ yield a broad spatial scale different from the observed functional connectivity (black dots vs. solid blue line). Shrinking both broad spatial scales (*σ*_*e,b*_ = 70 µm and *σ*_*i,b*_ = 40 µm) yields a more quantitative match of decay to zero (dot-dash blue line). Experimental data is represented as mean ± SEM (N = 160 ensembles). **g:** The spatial footprint of disynaptic connections using the anatomical data from Rossi et al.^22^ (*σ*_di_ ≈(147.31^2^ + 111.26^2^)^0.5^ = 180 µm) and the functional data from optogenetic perturbations (σ_di_ ≈(70^2^ + 40^2^)^0.5^ = 80 µm). **h:** When synaptic connectivity only followed a spatial wiring rule with no specificity in orientation space (that is, *h*_*α*_(*θ*) = 1), there is no difference in the recruited recurrent activity of iso-oriented (solid blue) vs. orthogonally oriented (dashed red) neurons.

Together, our experimental results demonstrate that the photoactivation of small ensembles results in highly local recurrent amplification with large amounts of surround suppression. Furthermore, our data suggest that the amplitude of this recurrent activity depends upon the precise spatial distribution and feature preference of the activated ensemble. We now seek to understand how these results arise mechanistically by developing a computational model of these targeted optogenetic perturbations (Methods). We started by investigating how connectivity principles in L2/3 recurrent circuits could explain why recurrent activation is extremely local while suppression dominates at larger distances from the stimulated ensemble. We wired the simulated circuit based on previously acquired connectivity data^22^ (Extended Data Fig. 9a–d) and modeled the dynamics of the population with a two-dimensional neural field model (ref. ^44^; Methods). Due to the modest size and strength of the optogenetic perturbations, we considered the network response as linear perturbations around a steady-state firing rate solution. Furthermore, because the experiments were performed in the absence of visual input, we could assume that the neurons in the network have a low gain response, implying that the effective connectivity strength is relatively weak. This allowed us to investigate the network perturbation via a synaptic pathway expansion with just a few terms^26,45^. Specifically, we considered monosynaptic and disynaptic excitatory connections (E→E and E→E→E) and disynaptic inhibitory connections (E→I→E; Fig. 5a).

After fitting the spatial components of the model (Methods), we are left with the following two free parameters that correspond to the strengths of these pathways: *w*_ee_ (the effective strength of E→E connections) and *w*_eie_ (the effective strength of the inhibitory pathway). We find that nearby activation and surround suppression arise for a variety of parameter values, with near identical shapes arising for fixed values of *w*_ee_/*w*_eie_. After fixing *w*_eie_ and varying *w*_ee_ for simplicity, we find that we can adjust both the zero crossing of this curve and the strength of the nearby activation (Fig. 5b). However, we see that this model is unable to pass through the experimentally observed data regime (Fig. 5b (striped, gray box) and Extended Data Fig. 9e; Methods). Specifically, when these parameters are adjusted to match the experimentally observed cross at ∼35 µm, the model fails to capture the relative strength of nearby activation to more distant suppression.

To capture this key detail, we reasoned that we needed to add an additional narrow spatial scale (<50 µm) to the model (Fig. 5c). This aligns with recent work^46^, which showed that such a narrow spatial wiring rule can also explain small columnar structures arising in L2/3 mouse V1 neurons that is not strictly a salt-and-pepper organization^47,48^. Adding in such a tight spatial component (that is, ${\kappa }$ >0; Methods) allowed the model to simultaneously capture both the nearby activation and the appropriate zero crossing between activation and suppression. However, there remains a quantitative difference in the rates that these curves return to baseline, which in the model is set by the broad spatial parameters *σ*_*e*,*b*_ and *σ*_*i*,*b*_ (Methods). These values were set to be in line with existing transsynaptic tracing connectivity studies^22^ and captured anatomical connectivity. Modestly reducing these broad spatial scales can quantitatively reproduce the experimental data (Extended Data Fig. 9f,g), suggesting that the functional connectivity of these neuronal projections is narrower than the estimated anatomical connectivity. For the remainder of this paper, we choose to focus on the qualitative matches between the data and the theoretical predictions, using the estimated anatomical connectivity data, while not fitting the model directly to the data.

With this computational model in hand, we now varied the spatial distribution of the activated ensembles, which yielded similar results as our experimental observations. Namely, as the spatial distribution ensemble of neurons becomes more compact, the level of surround suppression (at 50–150 μm) increases (Fig. 5d). However with the model, we have access to both the excitatory and inhibitory pathways activated as a result of these stimulations, which allows us to test our hypothesis that compact ensembles recruit stronger levels of suppression due to the convergence of excitatory activity onto inhibitory neurons (Fig. 2c). Specifically, we examined in the model the relative strength of the E→E and E→I→E pathways as a function of the spatial spread of the ensemble. We found that as the ensemble spread decreases, both synaptic pathways increase in magnitude, but the strength of the inhibitory pathway increases faster, leading to the observed effect (Fig. 5e; slope is ∼9% greater for the inhibitory pathway).

### Selective convergence onto inhibitory connections generates feature-dependent suppression

Up to now, we have used our computational model to shed light on the spatial wiring rules responsible for driving the recruitment of recurrent activity in response to stimulated ensemble with differential spatial configurations (that is, compact versus diffuse). We now turn our focus to gaining a deeper understanding of how the ensemble’s orientation preference influences its recurrent impact. We start by using the computational model to determine which features of the circuit connectivity are required to generate the peculiar switch from like-to-like activation to like-to-unlike suppression observed in the recurrent neurons lying closest to the stimulated ensemble (Fig. 3c). We considered the following three hypotheses: (1) the orientation dependence of recurrent inputs could emerge on their own based purely on spatial connectivity rules and salt-and-pepper orientation tuning, (2) like-to-like E→E connectivity but random E→I and I→E connectivity could explain it or (3) orientation specificity would be required in all of these pathways. In line with previous work, we assumed that the orientation preferences of individual neurons are inherited from feedforward projections and are randomly distributed in physical space.

**Fig. 6:**
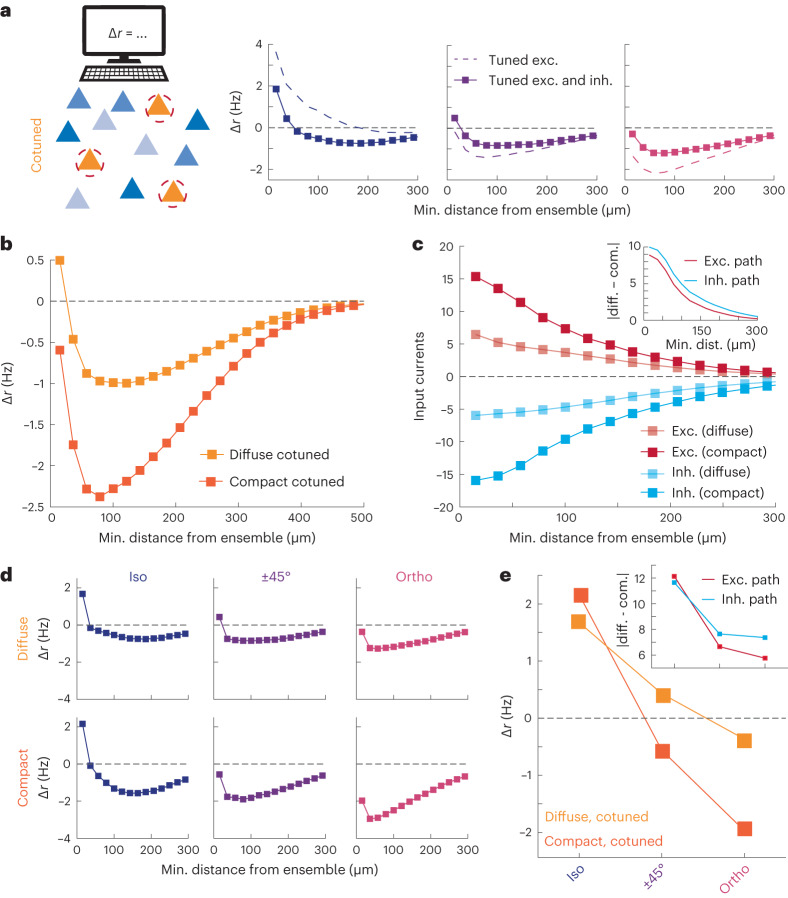
Network model with both spatial- and feature-based tuned connections wiring rules can recapitulate core experimental results. **a**, Same as Fig. 3c, except for the network model with (solid) and without (dashed) tuned E→I connections. **b**, Nontargeted cell responses in the network model as a function of their minimal distance to the ensemble according to the ensemble spread and tuning. **c**, The excitatory (red) and inhibitory (blue) input pathways for the spatially diffuse and compact cotuned ensembles. Inset, absolute difference of excitatory and inhibitory paths showing that while both pathways increase in magnitude for compact ensembles, the inhibitory pathway shows a larger increase. **d**, Nontargeted cell responses in the network model as a function of their minimal distance to the ensemble according to their relative tuning with the stimulated cotuned ensembles. **e**, Nontargeted cell responses from the first bin of panel **d** as a function of their relative tuning with the stimulated cotuned ensembles. Inset, absolute difference of excitatory and inhibitory pathways.

When synaptic connectivity only followed a spatial wiring rule with no specificity in orientation space, we found no difference in the recruited recurrent activity of iso-oriented versus orthogonally oriented neurons (Extended Data Fig. 9h), thus pure spatial rules are not sufficient on their own to explain the experimentally observed recurrent dynamics. Adding in like-to-like connectivity between excitatory neurons^22,49^ reproduced orientation preference-dependent effects, qualitatively similar to the experiment results (Fig. 6a, dashed). Specifically, cells that were iso-oriented to the photostimulated tuned ensemble showed activation, while those that are orthogonally orientated showed suppression. However, the model with this wiring scheme substantially overestimated iso-oriented activation and orthogonally oriented suppression at all distances and completely failed to capture the iso-oriented surround suppression beyond 50 μm. Finally, when the model incorporated like-to-like excitatory-to-inhibitory and inhibitory-to-excitatory connections, as recently suggested in ref. ^50^, it accurately reproduced the experimental data both qualitatively and quantitatively (Fig. 6a, solid). These results imply that feature-specific synaptic connectivity across all three synaptic pathways is essential to explain the space- and feature-dependence of recurrent cortical dynamics.

With our computational model now consisting of both the spatial- and feature-based wiring rules necessary to reproduce our core experimental findings, we use it to yield additional insights when both properties are considered in our stimulated ensemble (Fig. 4). Indeed, simulations showed that a diffuse, cotuned ensemble generated effects that are similar to previous results—nearby activation and suppression (Fig. 6b, orange curve), while compact, cotuned ensembles drove suppression across all distances (Fig. 6b, red curve). Decomposing this result into the direct excitatory and disynaptic inhibitory pathways, we found that although the compact ensemble recruits both more suppression and more activation than a diffuse ensemble (Fig. 6c), the suppression tends to dominate the net effects on firing rates. This observation illustrates an interesting tradeoff between the E→I→E inhibitory pathway and the excitatory pathways as one compresses the cotuned ensemble. Namely, nearby suppression replaces nearby activation as the ensemble shrinks in space.

**Extended Data Fig. 10:**
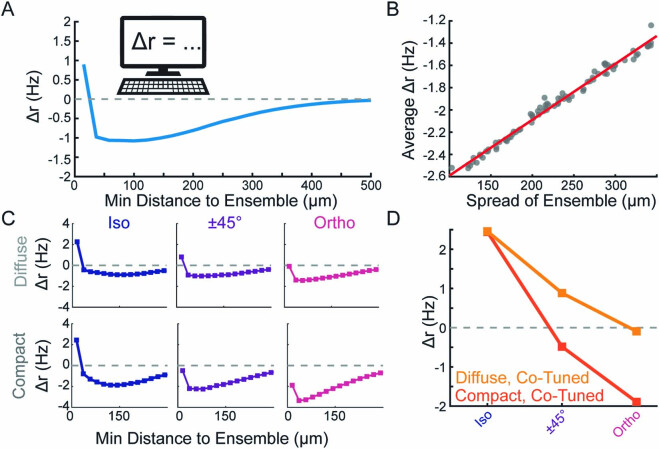
Final model with novel spatial- and feature-base rules captures all-core experimental results. The final model is capable of producing the all-core results of the paper, including **a:** nearby activation with surround suppression, **b:** a strong recruitment of suppression for tighter ensembles, **c:** non-targeted cell responses as a function of their minimal distance to the ensemble according to their relative tuning with the stimulated cotuned ensembles, and **d:** compact, cotuned ensembles sharpening the input signal locally more so than diffuse, cotuned ensembles. All panels use parameter values κ=0.045, *w*_*ee*_ = 0.0165, *w*_*eie*_ = −0.022.

After decomposing these effects based on the orientation preference of the nontargeted neurons, we find that the model predicts our experimental results—compact, cotuned ensembles differentially modulate the local signal more so than diffuse, cotuned ensembles (Fig. 6d,e). This final model, which also captures the data shown early in the study (Extended Data Fig. 10), explains that we can largely decompose the logic of recurrent cortical dynamics into a balance between the main two recurrent pathways (E→E and E→I→E). It further reveals that the stronger disynaptic recruitment of the inhibitory pathway via cells at non-iso orientations explains recurrent suppression of orthogonally tuned cortical ensembles (Fig. 6e, inset).

## Discussion

Recurrent activity in V1 could serve to amplify sensory input when signals are weak^2–6,11,12,51^, while driving competition among stimuli when signals are strong^7–10,52^. However, given the difficulty of isolating recurrent activity from feedforward or feedback activity, the fundamental role of recurrent activity in cortical computation remains untested. We studied the role of local recurrent activity by using 2P holographic optogenetics to selectively activate small ensembles of L2/3 cells without ongoing visual input. By leveraging our ability to design unique ensembles of L2/3 neurons, we systemically tested the role two fundamental axes (physical space and feature tuning) have in driving recurrent activity. Moreover, by combining this experimental approach with detailed computational modeling, we proposed a new wiring rule that depends on both the spatial and feature properties of cells.

We found that recurrent circuits could either amplify or suppress cortical activity depending on the spatial distribution and tuning of the presynaptic ensemble and the location and tuning of the postsynaptic cells. Most photostimulation patterns suppress cortical activity, but the sign, scale and magnitude of recurrent network modulation followed a specific logic. For example, we found that compact, cotuned ensembles largely drive suppression, while spatially distributed cotuned neurons drive amplification, but only of nearby neurons, otherwise, they likewise drive suppression. Taken together, our results demonstrate that the recurrent circuitry defined by feature space and physical space jointly determines the impact of recurrent circuits.

By choosing to focus on periods of no visual stimulus, we analyzed the recurrent dynamics in the absence of retinal/thalamic input. Although the evoked activity under these conditions may not precisely reflect naturally occurring population dynamics, it allows us to isolate the impact of purely recurrent synaptic interactions. Nonetheless, we can speculate that activated diffuse cotuned populations might relate to ensembles naturally activated by extended, oriented contours (for example, trees and branches), while compact untuned ensembles might be activated by local textures with diverse orientation content (for example, dense tangles of grass/weeds).

In a previous study, one-photon (1P) optogenetic stimulation of callosally projecting L2/3 excitatory neurons (via antidromic activation of their axons in the contralateral hemisphere) drove both activation and suppression of recurrent activity depending on the visual stimulus contrast^53^. Despite the elegance and simplicity of this approach, it had no control over the number, identity or location of the neurons stimulated, which would have masked the types of highly specific effects we observed here. Notably, we used relatively modest perturbations (∼10 action potentials added to ∼10 neurons) to avoid pushing the system out of its physiological operating range, and to make our computational models easier to interpret, our perturbations evoked only small deviations from the steady-state firing rate solution. Indeed, we were able to construct network simulations with few parameters that could accurately capture our initial experimental results and qualitatively predict further experimental outcomes.

Our experiments reveal the ‘inferred connectivity’ between neurons^10^, that is, the effect of neurons on each other, as opposed to monosynaptic contact as could be determined by transsynaptic tracing, or EM reconstruction^22,54–56^. Nonetheless, investigation of the model suggested that direct like-to-like connectivity not only between excitatory neurons but also between excitatory and inhibitory neurons was important for explaining the results. A narrow spatial scale of like-to-like E→E connectivity was likewise essential for accurately predicting the experimental data. Such a spatial scale was recently suggested^46^ to explain minicolumns (<50 µm) arising in mouse V1 (refs. ^47,48^). Similarly, single-cell stimulations^57^ (largely contained within a 100 µm radius) also suggest a microstructure of spatial connectivity on a similar scale.

Several recent studies have also used 2P optogenetics to probe functional connectivity in mouse V1. One recent study^25^ found that optogenetic stimulation of tuned ensembles in mouse V1 preferentially activated other cotuned cells, leading the resulting population activity to mimic that of a visual stimulus. In contrast, under most conditions in our data, suppression dominates, with recurrent excitation confined to a close distance to targeted cells. Several experimental and methodological differences could account for these seemingly distinct results. In short, the two studies asked different questions: the study discussed in ref. ^25^ focused on the behavior of a subset of cells putatively involved in perception, while the present study concentrated on the mean effect on the entire cell populations with minimal cell selection. Moreover, we deliberately made smaller perturbations of cotuned ensembles limited to about ten neurons, while this other study generally aimed to recruit larger numbers of neurons with apparently higher firing rates. Furthermore, we found that a critical predictor of the net effect of a stimulus was the spacing of the stimulated cells, a parameter not explored in the study discussed in ref. ^25^.

Generally consistent with our work, other 2P optogenetic studies in mouse V1 (refs. ^10,29^) largely observed suppression across the network, but also some nearby activation. While this better matches our results, these studies observed much more substantial like-to-like suppression, leading one of the studies^10^ to hypothesize that recurrent networks are primed for competition rather than amplification. However, there are three key differences between this study’s^10^ approach and the one we used here. First, they targeted single neurons rather than ensembles. Because most synapses in cortex are weak, the impact of adding spikes to one neuron could be substantially different than when activating ten. Second, they set a larger nearby exclusion criterion, potentially missing some of the dynamics that we found important. While this study did not find any linear interactions between feature preference, measured as signal correlation, and distance, this can be explained by the methodological differences between the two studies. Finally, the notion of compact versus diffuse nature of an ensemble, which we found to be a critical determinant of net impact, has no meaning for single-neuron perturbations.

Furthermore, both of these studies conducted their experiments while presenting visual stimuli to the animal. This may have been necessary to make it possible to measure the very small effects of single-neuron photostimulation^10^ or influence behavioral performance^29^, yet it also means that the network state was potentially dominated by nonrecurrent sources of input. We found the population suppression was unchanged by the mere presence of a visual stimulus; however, it remains possible that different visual stimuli uniquely affect optogenetically driven recurrent dynamics.

2P optogenetic studies are unique in the enormous potential parameter space of the perturbations. Some of these are readily under user control (such as the spacing or tuning of the targeted neurons, or the number and timing of pulses delivered to each neuron) and some are not (such as the exact number of neurons that are photostimulated and the exact timing of the evoked action potentials). Standardizing these parameters should aid in better comparison across studies. More generally, using an approach that ensures specific numbers and temporal patterns of the evoked spikes^58^ should obviate the need for matching these parameters.

With respect to our computational modeling, a similar study^26^ also made use of a linear rate-based model to explore the pathways driving recurrent circuit impacts during the photostimulation of a small number of neurons. Like this study, we found that the E→I pathway must be sufficiently strong and feature-specific to explain the large amount of suppression observed. However, this previous study largely focused on explaining how the optogenetic perturbation of a single cell influences recurrent activity. Here we were able to further develop a model that simultaneously incorporates space- and feature-based wiring rules due to the larger number of neurons in the stimulated ensemble. Specifically, by exploring different spatial distributions of the activated ensemble, we observed interesting trade-offs between like-to-like amplification and suppression on different spatial scales. Such ensemble geometries are simply not possible in single-cell perturbation experiments. While another computational study^27^ also examined the effects of stimulating a larger number of neurons, they only considered cotuned ensembles and did not vary their spatial distribution.

In addition to these modeling examples that investigate similar holographic perturbation experiments, there has been a wealth of broader modeling work done in the context of exploring E–I circuit mechanisms that enhance cortical computations^59,60^. Recent work^61^ studying the dynamics of mouse V1 investigated how a stimulus-response can be modulated by the context of the surround. Extending previous works^62,63^ on surround suppression to account for different interneuron subtypes (that is, parvalbumin- (PV), somatostatin- (SOM) and vasointestinal peptide-expressing (VIP) neurons), they identified the VIP→SOM disinhibitory circuit as the crucial pathway in driving the response to cross-oriented gratings. The study discussed in ref. ^64^ used a similar modeling framework to show how this canonical cortical disinhibitory circuit modulates the synchrony of gamma oscillations across space. While these models incorporated both feature- and spatial-based wiring rules, they did so in a binary manner, accounting only for interactions across iso- versus cross-preferred neurons and center versus surround. While this minimalistic approach provided valuable insight into the experiment mechanisms considered, this framework is limited in its ability to extrapolate to other experimental conditions. Our investigation, which includes connectivity rules in a spatially continuous modeling framework, provides a key stepping-stone as the field moves toward a more unified model of mouse V1 that can capture a wealth of experimental data across different stimulus inputs and brain states.

An important constraint to consider is that we, as with most calcium imaging studies, have relatively low temporal resolution. As such, we are unable to detect transient activity patterns, such as brief activation followed by slower suppression, and the model is under-constrained in this regime to make insightful predictions at these faster timescales. Here it was sufficient to solve a linear system for Δ*r* (that is, the perturbation of the system in response to the optogenetic stimulation) in steady state (Methods) due to the large analysis window. In future studies, the faster properties of GCaMP8f, for example, could enable advances in the modeling framework. Specifically, to capture a transient amplification and delayed suppression potentially revealed by faster GCaMP kinetics, the corresponding model would need to include temporal dynamics and nonlinearities^65^. Furthermore, the higher-resolution experimental data would provide the additional and necessary constraints to parameterize such rate- and spiking-based models. This compelling next step, both experimentally and computationally, will be able to further dissect the tradeoff between the recurrent E→E and E→I→E pathways we observed here.

Our combined in vivo and in silico interrogation of recurrent dynamics helps define an elementary logic for the impact of recurrent circuits on cortical activity. Beyond simply amplifying or suppressing activity, our findings show that the impact of recurrent circuits in L2/3 depends jointly on physical space and feature space. Our computational modeling makes clear, testable predictions about the underlying circuitry enabling these local computations. The richness in recurrent modulation we discovered here matches the sophisticated demands of processing complex images, such as occur naturally in the world. More generally, the principles revealed in our work may constitute an elemental neural syntax of cortical transformations by recurrent circuits.

## Methods

All experiments were performed in accordance with the guidelines and regulations of the ACUC of the University of California, Berkeley (protocol AUP-2014-10-6832-2).

### Mice

All calcium imaging experiments were performed in adult mice (2–12 months old) of both sexes expressing GCaMP6s in excitatory neurons via tetO-GCaMP6s (Jackson Laboratory, 024742) × Camk2a-tTA (Jackson Laboratory, 003010). We confirmed the selectivity of this approach using RNAscope (Extended Data Fig. 1). ChroME^18^ or ChroME2s^31^ was transfected via Adeno-Associated Virus (AAV). All constructs were bicistronically linked to a nuclear-localized mRuby3 used for targeting photostimulation. Excitatory specificity was ensured using either a cre-dependent AAV (Syn-ChroME; Addgene, 170161 or CAG-ChroME2s; Addgene, 170163) in an excitatory specific cre line (Emx1-Cre; Jackson Laboratory, 005628 or SepW1-Cre MGI:5519915) or a Tta-dependent AAV (Tre-ChroME or Tre-ChroME2s; Addgene, 170177) using the same Camk2-tTa source as above. In some cases, other cre lines (Jackson Laboratory, 017320 or Jackson Laboratory, 013044) were crossed to the tetO-GCaMP6s × Camk2-tTa line, with other cre-dependent AAV fluorophores/indicators, those results are not a part of this study. No difference was observed between any mouse preparation (Extended Data Fig. 5). Control mice had the same tetO-GCaMP6s × Camk2a-tTA without any viral injections. Mice were housed in cohorts of five or fewer in a reverse 12-h light/12-h dark cycle, with experiments occurring during the dark phase.

### Surgery

All experiments were performed in accordance with the guidelines and regulations of the Animal Care and Use Committee of the University of California, Berkeley. For head fixation during experiments, a small custom stainless-steel headplate was surgically implanted. Briefly, adult mice (P35–P50) were anesthetized with 2–3% isoflurane and mounted in a stereotaxic apparatus. Body temperature was monitored and maintained at 37 °C. The scalp was removed, the fascia retracted and the skull was lightly scored with a drill bit. Vetbond was applied to the skull surface, and the headplate was fixed to the skull with dental cement (Metabond). A fine-point marker was used to note the approximate location of bregma and the left V1 (2.7 mm lateral, 0 mm posterior to lambda). In total, 2–3 burr holes were drilled using a dental drill (Foredom) with a 0.24 mm drill bit (George Tiemann & Co.), and 200–300 nl of AAV was injected at 50 nl min^−1^, followed by a 5+ minute waiting period. A 3–3.5 mm region of the skull surrounding the marked V1 area was removed using the dental drill and/or a biopsy punch (Robbins Instruments). The window was replaced with three glass coverslips (two 3 mm and one 5 mm) and cemented into place with dental cement. Mice were given additional saline during surgery (0.9% NaCl (0.3 ml)). Mice received buprenorphine and meloxicam for pain management and dexamethasone to reduce brain swelling.

### Two-photon imaging and stimulation microscope

All in vivo experiments were performed using a setup capable of 3D-SHOT, as described previously^17,18,31,66^. The microscope is adapted on a movable objective microscope (Sutter Instrument) platform, with the following three combined optical paths: a 3D 2P photostimulation path, a fast resonant-galvo raster scanning 2P imaging path and a widefield 1P epifluorescence/IR-transmitted imaging path, merged by a polarizing beamsplitter before the microscope tube lens and objective. Imaging was performed with a Chameleon Ultra II (Coherent), and photostimulation was performed with a Monaco40 (Coherent). Temporal focusing of the photostimulation beam from the femtosecond fiber laser was achieved with a blazed holographic diffraction grating (Newport Corporation, R5000626767-19311). The beam was relayed through a rotating diffuser to randomize the phase pattern and expand the temporally focused beam to cover the area of the high-refresh-rate spatial light modulator (SLM; HSP1920-1064-HSP8-HB, 1920 × 1152 pixels; Meadowlark Optics). Holographic phase masks were calculated using the Gerchberg–Saxton algorithm and displayed on the SLM to generate multiple temporally focused spots in 2D or 3D positions of interest. The photostimulation path was then relayed into the imaging path with a polarizing beamsplitter placed immediately before the tube lens. As described in ref. ^18^, to limit imaging artifacts introduced by the photostimulation laser, the photostimulation laser was synchronized to the scan phase of the resonance galvos using an Arduino Mega (Arduino), gated to be only on the edges of every line scan.

### Calibration

Two-photon activation of cells requires very precise alignment of the stimulation and the imaging system throughout a large 3D volume. Most calibration procedures assume that individual imaging and stimulation planes are parallel and flat. However, certain optical elements and subtle misalignments of the microscope can add aberrations that introduce mistargeting errors, especially at the edges of the FOV. For this reason, we improved our previous calibration approaches^17,18,67^ with a new fully automated multiplexed 3D calibration, which accounts for arbitrary distortions in either the imaging or stimulation planes (Extended Data Fig. 2a–h). We confirmed that our system is able to deliver arbitrary powers to arbitrary locations in single and multitarget holograms. As expected, we found that multitarget holograms were less efficient than single-target holograms, that is, more light is lost to diffraction. But for holograms of three or more targets, the light intensity hitting a given target is not affected by the identity or number of other targets (Extended Data Fig. 2i). For this reason, in all subsequent experiments, we restrict holograms to contain at least three target cells.

### Holographic stimulation

Cells were targeted for stimulation based on the nuclear-localized mRuby signal bicistronically linked to the opsin. Only multitarget holograms of at least three targets were used. Putative opsin-positive cells were analyzed online using scanImage2019a (Vidrio) by collecting fluorescence scores around each automatically detected red nuclei. ROIs that were not holographically activatable were not included in further experiments. Online data were only used during the experiment and were not used in analyses.

To minimize the risk of off-target activation, we minimize the power used per cell by first performing a ‘power test’ on each cell. In groups of five cells at a time, we activated each cell with five 5 ms pulses of light at powers ranging from 12.5 to 100 mW per cell. We define the ‘stimmable power’ as the power in which we could elicit a significant calcium response in a given cell. ChroME, and its derivatives, are useful in that using excess power does not easily elicit more than one spike per 5 ms pulse^18,31,66^. Therefore, we multiply the stimmable power by 1.1 to 1.2 to ensure a more faithful response in each stimulated cell. Throughout the experiments, multitarget holograms are designed such that each cell receives a distinct power based on its stimmability and the diffraction efficiencies of each spot. We further restrict the analysis to exclude cells within 15 µm on the same plane or within 30 µm one plane away (30 µm spacing), as they have a risk of receiving off-target light.

To confirm our resolution, we obtained PPSFs in two separate experiments from a total of 26 matched cells. After the standard ‘power test,’ randomly selected sets of ten cells distributed throughout the FOV were driven as in a standard experiment. Holograms were digitally offset radially using 3 µm steps (range: −3 to 30 µm from aligned) and axially in 6 µm steps (range: −6 to 60 µm). Resulting fluorescence was fit with a Gaussian, aligned to the peak and the full-width half maximum (FWHM) was obtained.

To be conservative, we excluded cells that were closer than 15 µm from a stimulated target. This exclusion zone is larger than any PPSF FWHM that we observed (Fig. 1d). Similarly, we could not detect any differences in the responses of opsin-positive or opsin-negative cells beyond 10 µm from a stimulated cell (Extended Data Fig. 3).

### Calcium imaging

All recordings were performed in L2/3 imaging three 800 × 800 µm planes, spaced 30 µm apart, at 5.2–6.2 Hz with <75 mW (920 nm) laser light (Coherent Chameleon Ultra II) using a resonant-galvo system. Images were acquired using ScanImage (Vidrio) with custom stimulation control software. During recordings, animals are on a running wheel and their run speed is recorded.

Visual stimuli were presented on a 2,048 × 1,536 Retina iPad LCD display (Adafruit Industries) placed 10 cm from the mouse. The monitor backlight was synchronized with the galvos such that it came on only during the turnaround time, so that light from the monitor did not contaminate 2P imaging. Visual stimuli were created and presented with custom MATLAB code and Psychophysics ToolBox. Drifting gratings (50 visual degrees, 1 Hz, 0.08 cycles per degree, 100% contrast) of different orientations were randomly presented for 1 s in each trial and interleaved with a gray-screen (blank) condition. Neurons with significantly different responses to visual stimuli (*P* < 0.05, ANOVA) were considered as visually responsive. For the subset of experiments with optogenetic stimulation delivered alongside contrast noise stimuli, full-screen (50 visual degrees) Gaussian contrast noise stimuli were presented at varying contrast (0%, 1%, 4%, 10%, 40% and 100%)^68^. For these experiments, visual stimulation was triggered 200 ms before optogenetic stimulation to account for delays in visual stimulus onset. The visual stimulus remained on for the duration of the holographic stimulus (1 s).

### Online analysis

Tuning curves and responses to photostimulation were calculated during the experiment, using a custom online implantation of CaImAn OnACID (v1.8.8)^69^ to perform rigid motion correction and seeded source extraction (https://github.com/willyh101/live2p). Preferred orientation (PO) was calculated as the maximum mean response to oriented gratings, orthogonal orientation (OO) as the mean response to a grating of orthogonal orientation, and orientation selectivity index (OSI) was calculated as (PO − OO)/(PO + OO).

### Discrete optimizer

In some experiments, it was difficult to manually identify the optimal targets to create distinct ensembles that fit certain criteria, such as close and cotuned ensembles. To overcome this challenge, we wrote a custom discrete optimizer. This optimizer selects groups of targets to stimulate from a database of eligible cells to minimize a custom cost function. As ‘cells to include’ is a discrete operation, each step of our optimizer swaps one or more cells before evaluating the cost function and continuing. For a given experimental day, we optimized for 3–20 ensemble of ten cells that (1) were maximally distinct from each other, (2) minimized the number of times individual cells were included in different holograms, (3) prioritized cells activated with low light powers, (4) prioritized visually responsive cells, (5) avoided instances where two cells in the same ensemble were within 30 µm of each other, (6) spread out cells within an ensemble, (7) fit the desired spatial rules (for example were spatially compact versus spread out), (8) fit the desired ensemble tuning (that is, ensemble OSI) and (9) fit the desired mean selectivity (that is, OSI of ensemble members was high for cotuned ensembles or low for untuned ensembles).

### Offline analysis

Tiff files were motion corrected, cell sources (aka pixel masks) were determined and source fluorescence was extracted using suite2p (version released summer 2017)^70^. Pixel masks were manually categorized as ‘cells’ or ‘not cells,’ and only ‘cells’ were included for analysis. For Δ*F*/*F* calculation, each cell’s detected fluorescence was first neuropil subtracted. The average fluorescence of an annulus (not containing another cell) of up to 350 pixels was considered neuropil. For all figures except Extended Data Fig. 6f,g, a neuropil coefficient (*c*) was calculated for each cell as described in ref. ^70^, and the final fluorescence was calculated as *F* = *F*_cell_ – *c* × *F*_neuropil_. In Extended Data Fig. 6f,g, a fixed neuropil coefficient was used for every cell, as determined in that figure. *F*_0_, the ‘baseline’ fluorescence, was calculated with a moving average of the tenth percentile of a 1,000 frame window (∼3 min); this moving average is used to correct for very slow drift in imaging conditions. Δ*F*/*F* is calculated as (*F* − *F*_0_)/*F*_0_.

Not all putative cells identified via red nuclei and/or online analysis were recovered by suite2p. This ‘nonmatched’ population could be caused by a variety of sources, including errors in the online initial detection algorithms, errors in suit2p’s recovery and potentially errors in the manual ‘cell’ versus ‘not cell’ determination. If too many cells of an ensemble did not match, that ensemble was excluded (Exclusion criteria). When calculating the distance to a target or spread of an ensemble, the targeted rather than recovered sets of coordinates are used.

The minimum distance to a target was defined for each cell as the minimum distance to any attempted target, regardless of whether that target ‘matched’ to a suite2p detected cell.

The spread of an ensemble was calculated as the mean pairwise distance between the center of mass of each target of an ensemble calculated in 3D. A close ensemble is defined as having a mean pairwise distance <200 µm, whereas a far apart ensemble has a mean distance >200 µm.

Tuning curves and OSIs were recalculated offline data for subsequent analysis. Ensemble OSI is defined as the OSI of the mean tuning curve from cells used in an ensemble. Mean OSI is the arithmetic mean of the OSIs from each ensemble. Cotuned ensembles are defined as ensembles with an ensemble OSI > 0.7 and a mean OSI > 0.5; untuned ensembles are defined as ensembles with an ensemble OSI < 0.3 and a mean OSI < 0.5.

### Statistics and reproducibility

Throughout this work, nonparametric two-sided tests are used except where noted. Individual FOVs may come from the same mouse but comprise a different area or plane, and thus consist of different neurons. We consider the effects of different ensembles as the appropriate level of analysis but report the hierarchical nature of the data. No statistical method was used to predetermine the sample size. Holograms were randomly assigned and randomly interleaved during data collection blind to the experimenter. Batch analysis was performed across experimental conditions, thus blinded during data analysis.

### Exclusion criteria

Trials were excluded if (1) the animal ran more than 6 cm s^−1^, (2) 50% or more of the targeted cells failed to respond when driven (to at least 0.25 *z*-scored fluorescence above baseline) or (3) registration of the FOV indicates that the brain shifted more than 4.7 µm (3 pixels), indicating a miss.

Cells were excluded from a given trial if (1) they were located in an off-target region (15 µm radially from a targeted cell or 30 µm radially from a cell one plane away), (2) they had been stimulated in the immediate preceding trial, (3) they were occluded by the stimulation artifact or (4) the cell was categorized as ‘not cell’ or not detected via the suite2p process.

Ensembles were excluded from analysis if (1) more than 33% of the targeted cells were not detected via suite2p, (2) more than 50% of attempted stimulation trials failed (note only successful trials are included) or (4) had fewer than ten repetitions for either the baseline or (5) stimulation conditions.

FOVs were excluded from analysis if (1) fewer than 5% of cells were visually responsive, (2) more than 50% of trials occurred while the mouse was running or (3) fewer than 250 total cells were detected by suite2p.

### Determining opsin-negative cells

Opsin-positive and opsin-negative cells were identified in SepW1-Cre × CamK2a-tTA × tetO-GCaMP6s mice injected with AAV-CAG-DIO-ChroME2s-P2A-H2B-mRuby3, as described above. In addition to the typical imaging procedures, a structural image of the FOV at 1,020 nm was taken at the start of an experiment to identify and quantify the brightness of the nuclear mRuby3. As window preparations and imaging conditions could vary between days, the mRuby3 brightness was considered a relative measure. For each FOV, the top 20% brightest red nuclei were defined as opsin-positive, while the 30% dimmest were considered opsin-negative. Opsin-negative cells often scored low integer values fluorescence counts, with many cells receiving equal scores, thus in some recordings more than 30% of cells were included.

### Mathematical model

We consider a two-dimensional neural field model of the form

$${\tau }_{\alpha }\frac{\partial {r}_{\alpha }}{\partial t}=-{r}_{\alpha }+{\phi }_{\alpha }\left({\,j}_{\mathrm{\alpha e}}* {r}_{e}+{j}_{\mathrm{\alpha i}}* {r}_{i}+{\mu }_{\alpha }\right),$$

where * denotes a two-dimensional convolution in space, *α* = *e, i* and (*x*, *y*) $\in$ [0,1400] × [0,1400] µm box with periodic boundary conditions (modified from ref. ^44^). Because the animal is viewing a gray screen, there exists a uniform steady state for this system, *r*_ss_. We then make use of the fact that the perturbation to the system is relatively weak, and as a result, we can approximate it as a linear perturbation around *r*_ss_. Linearizing the system yields

$$T\frac{\partial \Delta {{r}}}{\partial t}=\left(-I+W\right)* \Delta {r}{,}$$

where

$${W}_{\alpha \beta }={g}_{\alpha }\cdot {j}_{\alpha \beta },$$

with *g*_*α*_ being the gain set by the steady state of the system (that is, ${g}_{{\alpha }}={{\phi }}_{{\alpha }}^{{\prime} }|_{\mathrm{ss}}$). The optogenetic stimulation is then modeled by considering a perturbation of the form

$$T\frac{\partial \Delta {{r}}}{\partial t}=\left(-I+W\right)* \left(\Delta {{r}}+{{{r}}}_{{stim}}\right),$$

where *r*_*stim*_(*x*, *y*) = 10·*δ*(*x* − *x*_*i*_) *δ* (*y* − *y*_*i*_) at the ten stimulated locations denoted by (*x*_*i*_, *y*_*i*_). Transforming the system into Fourier space, we can solve for Δ*r* in steady state

$$\Delta \widetilde{{{r}}}={\left(I-\widetilde{W}\right)}^{-1}\cdot \widetilde{W}\cdot {\widetilde{{{r}}}}_{{stim}}.$$

We can perform a matrix expansion of this inverse as long as the spectral radius of *W̃* is less than one. Because V1 is in a low gain state while the animal is viewing a gray screen, the effective connection strength is very weak (that is, ${g}_{{\alpha }}\ll 1$) placing us within this regime. Performing this expansion yields

$$\Delta \widetilde{{{r}}}=\left(I+\widetilde{W}+{\widetilde{W}}^{\,2}+\ldots \right)\cdot \widetilde{W}\cdot {\widetilde{{{r}}}}_{{stim}}.$$

Finally, after taking the inverse Fourier transform, we find that

$$\Delta {{r}}=\left(W+W* W+{W}^{*3}+\ldots \right)* {{{r}}}_{{stim}}.$$

We can again make use of the weak effective connectivity to drop the higher-ordered terms and write

$$\Delta {{r}}\approx \left(W+W* W\right)* {{{r}}}_{{stim}}.$$

Because we are only stimulating and recording from excitatory neurons, we can write this approximation as a sum of monosynaptic and disynaptic excitatory terms and a disynaptic inhibitory pathway

$$\Delta {r}_\mathrm{e}\approx \left({W}_{\mathrm{ee}}+{W}_{\mathrm{ee}}* {W}_{\mathrm{ee}}\right)* {r}_{{stim}}+{W}_{\mathrm{ei}}* {W}_{\mathrm{ie}}* {r}_{{stim}}.$$

While we consider connectivity rules that depend on both space and feature, we assume that these components are independent. This allows us to write the strength of connection between neurons at coordinates (*x*_1_, *y*_1_) and (*x*_2_, *y*_2_) with feature preference *θ*_1_ and *θ*_2_, respectively as

$$\begin{array}{l}{W}_{\alpha \beta }\left({x}_{1},{y}_{1},{\theta }_{1}{\rm{;}}\,{x}_{2},{y}_{2},{\theta }_{2}\right)\\={w}_{\alpha \beta }\cdot g\left(\sqrt{{\left({x}_{1}-{x}_{2}\right)}^{2}+{\left({y}_{1}-{y}_{2}\right)}^{2}}{\rm{;}}{\sigma }_{\beta },{\kappa }_{\beta }\right)\cdot {h}_{\beta }\left({\theta }_{1}-{\theta }_{2}\right)/z\end{array}$$

where *z* is a normalization factor and *r*^2^ = (*x*_1_ − *x*_2_)^2^ + (*y*_1_ − *y*_2_)^2^ is the distance between the two locations, adjusted accordingly to account for the periodic boundary. The spatial dependence is given by the following sum of Gaussian distributions:

$$g\left(r{\rm{;}}{\sigma }_{\alpha },{\kappa }_{\alpha }\right)=\frac{\left(1-{\kappa }_{\alpha }\right)}{2\pi {\sigma }_{\alpha ,b}^{2}}{\cdot e}^{-{r}^{2}/\left(2{\sigma }_{\alpha ,b}^{2}\right)}+\frac{{\kappa }_{\alpha }}{2\pi {\sigma }_{\alpha ,n}^{2}}{\cdot e}^{-{r}^{2}/\left(2{\sigma }_{\alpha ,n}^{2}\right)},$$

where *σ*_*α*,*b*_ refers to the broad spatial component, *σ*_*α*,*n*_ corresponds to the narrow spatial component and *κ*_*α*_ is the relative weight of each of term. The broad spatial components for the outgoing excitatory and inhibitory connections are based on the data from ref. ^22^ (Extended Data Fig. 9a,b). The parameters of the narrow component, *σ*_*α*,*n*_ and *κ*_*α*_ are adjusted from ref. ^46^, chosen to capture the nearby excitation observed in Fig. 2a and Extended Data Fig. 9e. Furthermore, the boundary of the experimentally observed data regime box used in Fig. 5b was found by fitting experimental data for different bin widths (5–20 μm) to the function

$$f\left(d\right)={A}_{1}{e}^{{\left(d/{\sigma }_{1}\right)}^{2}}+{A}_{2}{e}^{{\left(d/{\sigma }_{2}\right)}^{2}},$$

Solving for the zero crossing and maximum activation/maximum suppression and then taking the boundary to be the smallest rectangle that includes the values for all bin widths.

The models without feature-based connectivity take *h*_*α*_(*θ*) = 1. Otherwise, it takes the form

$${h}_{\alpha }\left(\theta \right)={r}_{0}+{r}_{p}\cdot {e}^{{-\theta }^{2}/\left(2{\sigma }_{\alpha \beta }^{2}\right)},$$

where *θ* ∈ [0, 90]. The parameters for feature-based excitatory connections are also based on the data from ref. ^22^ (Extended Data Fig. 9c,d), whereas the inhibitory connections are adjusted to best match the observed like-to-like suppression seen in Fig. 3c. After using the available data, the free parameters are the effective strength of excitatory connections (*w*_ee_), the effective strength of the inhibitory pathway (*w*_eie_ = *w*_ei_·*w*_ie_), the narrow spatial components parameters and the feature-base rules of the inhibitory connections.

### RNA in situ hybridization

Brain was collected from a 6-month-old tetO-GCaMP6s^+/+^ CamK2a-tTA^+/−^ female, embedded in optimal cutting temperature compound (Tissue-Tek) and frozen on dry ice within 5 min of tissue collection. Tissue blocks were cut into 10 µm sections using a cryostat. RNAscope was performed on the sections according to the manufacturer’s instructions (RNAscope Fluorescent Multiplex Kit; Advanced Cell Diagnostics). Probes used were Mm-GCaMP6s-O1, Mm-Slc32a1-C2 and Mm-Slc17a7-C3. RTU DAPI was used to stain cell nuclei, and slides were mounted using Vectashield mounting medium (Vector Laboratories). Images were collected using LSM 880 NLO AxioExaminer confocal microscope (Zeiss) and processed using ZEN lite (Zeiss). For analysis, 300 cells with positive DAPI staining from five slices were counted in cortical L2/3 and positive/negative staining of each probe was recorded for each cell. Cell with less than ten dots per probe was presumed negative for the respective RNA.

### Reporting summary

Further information on research design is available in the Nature Portfolio Reporting Summary linked to this article.

## Online content

Any methods, additional references, Nature Portfolio reporting summaries, source data, extended data, supplementary information, acknowledgements, peer review information; details of author contributions and competing interests; and statements of data and code availability are available at 10.1038/s41593-023-01510-5.

### Supplementary information

Reporting Summary.

## Data Availability

The compressed data to reproduce the key figures of the paper can be found on the GitHub repository (https://github.com/gregoryhandy/Logic_of_Recurrent_Circuits).

## Code availability

Code to reproduce the key figures of the paper can be found on the GitHub repository (https://github.com/gregoryhandy/Logic_of_Recurrent_Circuits). Additional code used for analysis can be found at: https://github.com/willyh101/100spikesAnalysis. Code used for online analysis can be found at https://github.com/willyh101/live2p.
