# Supplementary material for: The logic of recurrent circuits in the primary visual cortex
Source: Nat Neurosci. 2024 Jan 3;27(1):137–47. doi: 10.1038/s41593-023-01510-5 (PMC10774145; doi:10.1038/s41593-023-01510-5)
Supplement: Supplementary file 1 — Reporting Summary. [file 41593_2023_1510_MOESM1_ESM.pdf]

nature portfolio

Corresponding author(s): Ian Oldenburg, Gregory Handy

Last updated by author(s): Oct 6, 2023

Reporting Summary

Nature Portfolio wishes to improve the reproducibility of the work that we publish. This form provides structure for consistency and transparency in reporting. For further information on Nature Portfolio policies, see our Editorial Policies and the Editorial Policy Checklist.

Statistics

For all statistical analyses, confirm that the following items are present in the figure legend, table legend, main text, or Methods section.

n/a	Confirmed	
☐	☒	The exact sample size (*n*) for each experimental group/condition, given as a discrete number and unit of measurement
☐	☒	A statement on whether measurements were taken from distinct samples or whether the same sample was measured repeatedly
☐	☒	The statistical test(s) used AND whether they are one- or two-sided<br>*Only common tests should be described solely by name; describe more complex techniques in the Methods section.*
☒	☐	A description of all covariates tested
☐	☒	A description of any assumptions or corrections, such as tests of normality and adjustment for multiple comparisons
☐	☒	A full description of the statistical parameters including central tendency (e.g. means) or other basic estimates (e.g. regression coefficient) AND variation (e.g. standard deviation) or associated estimates of uncertainty (e.g. confidence intervals)
☐	☒	For null hypothesis testing, the test statistic (e.g. *F*, *t*, *r*) with confidence intervals, effect sizes, degrees of freedom and *P* value noted<br>*Give P values as exact values whenever suitable.*
☒	☐	For Bayesian analysis, information on the choice of priors and Markov chain Monte Carlo settings
☐	☒	For hierarchical and complex designs, identification of the appropriate level for tests and full reporting of outcomes
☐	☒	Estimates of effect sizes (e.g. Cohen's *d*, Pearson's *r*), indicating how they were calculated

Our web collection on statistics for biologists contains articles on many of the points above.

Software and code

Policy information about availability of computer code

Data collection	Data was collected using ScanImage2019a
Data analysis	Data was processed and analyzed using open source code suites CaiMan (v,1.8.8) and Suite2P (vs 2017), as well as custom algorithms and scripts. Code to reproduce the key figures of the paper can be found on the GitHub repository: https://github.com/gregoryhandy/Logic_of_Recurrent_Circuits. Additional code used for analysis can be found at: https://github.com/willyh101/100spikesAnalysis. Code used for online analysis can be found at https://github.com/willyh101/live2p.

For manuscripts utilizing custom algorithms or software that are central to the research but not yet described in published literature, software must be made available to editors and reviewers. We strongly encourage code deposition in a community repository (e.g. GitHub). See the Nature Portfolio guidelines for submitting code & software for further information.

Data

Policy information about availability of data

All manuscripts must include a data availability statement. This statement should provide the following information, where applicable:

- Accession codes, unique identifiers, or web links for publicly available datasets
- A description of any restrictions on data availability
- For clinical datasets or third party data, please ensure that the statement adheres to our policy

The compressed data to reproduce the key figures of the paper can be found on the GitHub repository: https://github.com/gregoryhandy/

Logic_of_Recurrent_Circuits

Research involving human participants, their data, or biological material

Policy information about studies with human participants or human data. See also policy information about sex, gender (identity/presentation), and sexual orientation and race, ethnicity and racism.

Reporting on sex and gender	N/A
Reporting on race, ethnicity, or other socially relevant groupings	N/A
Population characteristics	N/A
Recruitment	N/A
Ethics oversight	N/A

Note that full information on the approval of the study protocol must also be provided in the manuscript.

Field-specific reporting

Please select the one below that is the best fit for your research. If you are not sure, read the appropriate sections before making your selection.

☒ Life sciences ☐ Behavioural & social sciences ☐ Ecological, evolutionary & environmental sciences

For a reference copy of the document with all sections, see nature.com/documents/nr-reporting-summary-flat.pdf

Life sciences study design

All studies must disclose on these points even when the disclosure is negative.

Sample size	No a priori sample size calculation was performed. Based on preliminary experiments, we determined that 10-15 ensembles per condition were sufficient to resolve our effects. Trial to trial fluctuations were a greater noise source, so we performed 15-25+ trials per condition.
Data exclusions	Exclusion criteria is explained in the manuscript: Trials were excluded if (1) the animal ran more than 6 cm/s, (2) 50% or more of the targeted cells failed to respond when driven (to at least 0.25 Z-scored Fluorescence above baseline), or (3) registration of the field of view indicates that the brain shifted more than 4.7µm (3 pixels), indicating a miss. Cells were excluded from a given trial if (1) they were located in an off target region (15µm radially from a targeted cell, or 30µm radially from a cell one plane away), (2) they had been stimulated in the immediate preceding trial, (3) they were occluded by the stimulation artifact, or (4) the cell was categorized as 'not cell' or not detected via the suite2p process. Ensembles were excluded from analysis if (1) more than 33% of the targeted cells were not detected via suite2p, (2) more than 50% of attempted stimulation trials failed (note only successful trails are included), or had fewer than 10 repetitions for either the baseline (4) or stimulation (5) conditions. Fields of view were excluded from analysis if (1) fewer than 5% of cells were visually responsive, (2) more than 50% of trials were occurred while the mouse was running, or (3) fewer than 250 total cells were detected by suite2p.
Replication	Data was replicated and is reproducible. Each condition is reported as the average of ~20 trials, which in general appear consistent over time. Furthermore the 160 unique ensemble perturbations that make up this study were generated from a total of 13 Mice which each independently support the primary findings. Furthermore the primary data that makes up this dataset was collected over a 3 year period. Analyzing any given year alone comes to the same conclusion as the complete dataset.
Randomization	Perturbations were randomized on a trial by trial basis. Every animal in this study underwent similar experimental procedure. Cells were divided into different ensembles (i.e. experimental groups) by random assignment after filtering for the desired properties (see methods).
Blinding	Holograms were randomly assigned and randomly interleaved during data collection blind to the experimenter. Batch analysis was performed across experimental condition, thus blinded during data analysis.

Reporting for specific materials, systems and methods

We require information from authors about some types of materials, experimental systems and methods used in many studies. Here, indicate whether each material, system or method listed is relevant to your study. If you are not sure if a list item applies to your research, read the appropriate section before selecting a response.

Materials & experimental systems

n/a	Involved in the study
☒	☐ Antibodies
☒	☐ Eukaryotic cell lines
☒	☐ Palaeontology and archaeology
☐	☒ Animals and other organisms
☒	☐ Clinical data
☒	☐ Dual use research of concern
☒	☐ Plants

Methods

n/a	Involved in the study
☒	☐ ChIP-seq
☒	☐ Flow cytometry
☒	☐ MRI-based neuroimaging

Animals and other research organisms

Policy information about studies involving animals; ARRIVE guidelines recommended for reporting animal research, and Sex and Gender in Research

Laboratory animals	All calcium imaging experiments were performed in adult mice (2-12 months old) of both sexes expressing GCaMP6s in excitatory neurons via tetO-GCaMP6s (Jax #024742) x Camk2a-tTA (Jax #003010). In some cases, other cre lines (Jax #017320, or Jax #013044) were crossed to the tetO-GCaMP6s x Camk2-tTa line, with other cre dependent AAV fluorophores/indicators, those results are not a part of this study. Mice were housed according to UC Berkeley's OLAC's standard of care (73F, 53% Humidity)
Wild animals	no wild animals were used in this study
Reporting on sex	6/13 Experimental Mice were female. No difference was observed based on sex. An Extended Data Fig reports the sex of each mouse.
Field-collected samples	no field collected samples were used in this study.
Ethics oversight	All experiments were performed in accordance with the guidelines and regulations of the ACUC of the University of California, Berkeley. Protocol #AUP-2014-10-6832-2.

Note that full information on the approval of the study protocol must also be provided in the manuscript.
